# Zebrafish model for studying vascular development and vascular-related disease

**DOI:** 10.3389/fcell.2026.1872737

**Published:** 2026-08-21

**Authors:** Hadeel T. Zedan, Fatma H. Ali, Himanshu Gaur, Ramani Ramchandran, Huseyin C. Yalcin

**Affiliations:** 1 Biomedical Research Center, Qatar University, QU Health, Doha, Qatar; 2 College of Health Sciences, Member of QU Health, Qatar University, Doha, Qatar; 3 Department of Pediatrics, Division of Neonatology, Children’s Research Institute, Medical College of Wisconsin, Milwaukee, WI, United States; 4 Department of Mechanical and Industrial Engineering, Qatar University, Doha, Qatar

**Keywords:** angiogenesis, cardiovascular disease, disease modeling, vascular development, zebrafish

## Abstract

Zebrafish (*Danio rerio*) is a powerful vertebrate animal model system for cardiovascular research. Their small size, optical transparency, fecundity, rapid development, remarkable regenerative capacity, and the availability of numerous fluorescent transgenic lines have made them an excellent experimental model for studying vascular development and disease. There is a high degree of conservation in the anatomy and physiology of the zebrafish vascular system relative to other vertebrates, thereby enabling zebrafish to model human vascular pathophysiology. Questions concerning genetic vascular disorders, including endothelial dysfunction, flow-induced alterations, lipid metabolism, atherosclerosis, and tumor angiogenesis, have been extensively studied in zebrafish. In this review, we describe the contribution of the zebrafish system to the current understanding of blood vessel formation and the use of zebrafish for modelling human diseases caused by dysregulated vasculatures. We also summarize the techniques, methods, and transgenic lines used to image and assess zebrafish vasculature and to study its role in the development and pathogenesis of cardiovascular conditions.

## 1 Introduction

The zebrafish, a small tropical freshwater fish from the Ganges River in India, has been extensively used in several fields of biological and medical research, particularly in studies related to developmental biology (Streisinger et al., 1981). Zebrafish (*Danio rerio*) has emerged as an important vertebrate animal model for studying vascular development in intact animals (Gore et al., 2012). When compared to other vertebrate models, critical pathways involved in vascular development and physiology are highly conserved in Zebrafish (Asnani and Peterson, 2014; Stein et al., 2007). In addition, the basic vascular anatomy is conserved between zebrafish and humans. This has allowed basic vascular biology findings to be translated, informing potential molecular mechanisms that may have gone awry in human conditions (Isogai et al., 2001). The zebrafish model offers unique advantages for studying vasculature development, including the embryos’ and larvae’s optical transparency, which enables easy visualization and high-resolution imaging of the heart and vasculature (McKinney and Weinstein, 2008). In addition, zebrafish embryos develop externally to the mother, making them easily accessible for collection and manipulation (Gore et al., 2012). Further, the small size of zebrafish embryos allows them to receive sufficient oxygen via passive diffusion. This allows other organs and tissues to continue developing normally for 7 days post-fertilization (dpf), even without a functional cardiovascular system (Gut et al., 2017). This also makes them well-suited to examining the role of blood flow in vascular development. Several vascular mutants (spontaneous mutations or experimentally induced mutations) are available that show specific vascular patterning defects (Stainier and Fishman, 1994; Covassin et al., 2009). The zebrafish has a single closed circulatory system, which is one of the first organ systems to develop and become functional during embryogenesis (Isogai et al., 2001). Zebrafish embryos develop rapidly, and the two-chambered heart is formed by 24 h post-fertilization (hpf), along with axial vessel formation (dorsal aorta and the posterior cardinal vein) (Eriksson and Löfberg, 2000). The zebrafish embryonic vascular system is formed through two successive morphogenetic processes: vasculogenesis and angiogenesis. Vasculogenesis describes the *in situ* aggregation of angioblasts into a blood vessel, while angiogenesis describes the sprouting of new vessels from pre-existing ones (Ellertsdóttir et al., 2010).

Establishing robust and reliable *in vivo* models is crucial for understanding complex physiological processes and the molecular and cellular mechanisms underlying the formation of a functionally active vascular network. Selecting appropriate experimental models of human disease is also critical for studying specific aspects of disease pathology and for developing therapeutic strategies that promote or inhibit vessel growth. In that regard, zebrafish have already provided fundamental insights into cardiovascular development and have helped dissect the molecular mechanisms underlying vascular network formation (McKinney and Weinstein, 2008; Isogai et al., 2009; Weinstein et al., 1995).

**FIGURE 1:**
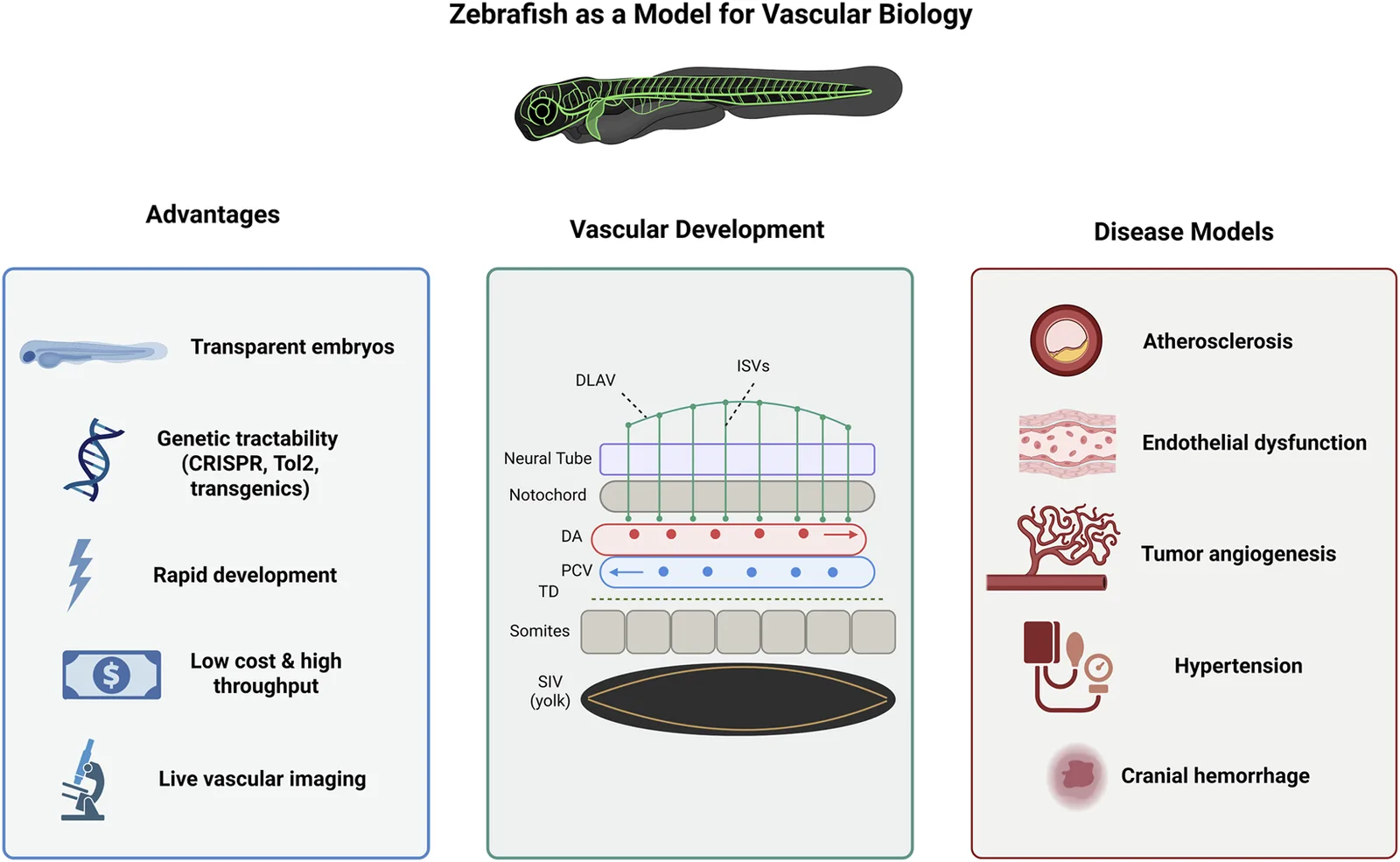
The utility of zebrafish as a vertebrate model for studying vascular development and vascular-related diseases.

In this review, we highlight the importance of zebrafish as an animal model for investigating vascular development and its role in disease. We also summarize the imaging techniques and transgenic lines used to examine zebrafish vasculature, and their contributions to our understanding of normal vascular development and its perturbation in disease. An overview of the topics covered in this review is presented in Figure 1.

## 2 Zebrafish vascular development

**FIGURE 2:**
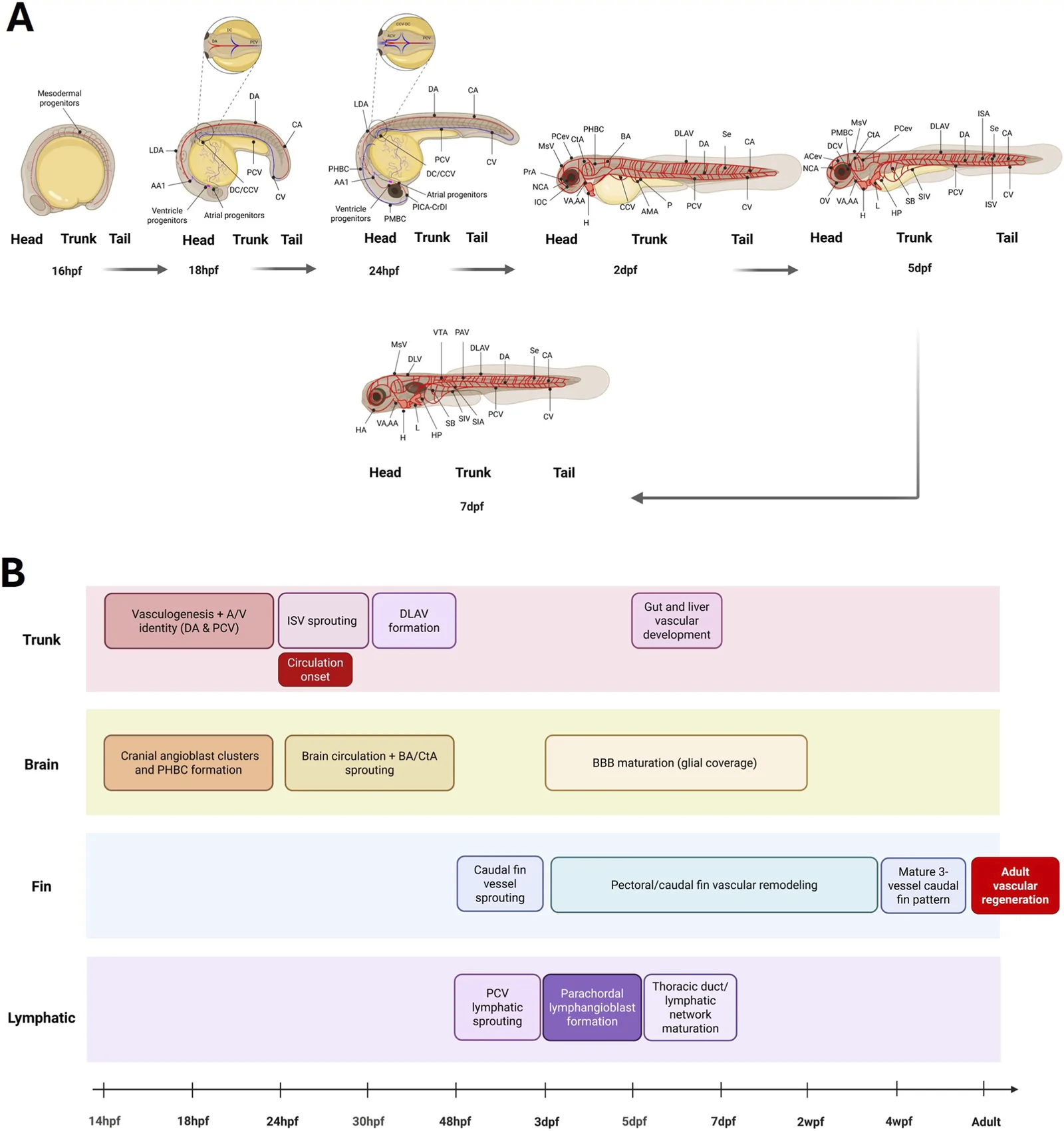
Overview of zebrafish vascular and lymphatic development. **(A)** Vascular development of zebrafish from 16 h post-fertilization (hpf) to 7 days post-fertilization (dpf). Mesodermal progenitors differentiate into endothelial cells beginning at 16 hpf, forming the dorsal aorta (DA) and posterior cardinal vein (PCV) and establishing a single primitive circulatory loop by 18 hpf; circulation initiates around 24–26 hpf. Over the following days, the cranial and trunk vasculature progressively elaborate into the mature vessel network shown, including the intersomitic vessels (ISVs), dorsal longitudinal anastomotic vessel (DLAV), and the primordial midbrain and hindbrain channels (PMBC, PHBC) and their derivatives. Abbreviations: AA1, mandibular aortic arch; ACeV, anterior cerebral vein; AMA, anterior mesenteric artery; BA, basilar artery; CA, caudal artery; CCV, common cardinal vein; CrDI, cranial division of the internal carotid artery; CV, caudal vein; DA, dorsal aorta; DC, duct of Cuvier; DCV, dorsal ciliary vein; DLAV, dorsal longitudinal anastomotic vessel; DLV, dorsal longitudinal vein; H, heart; HA, hypobranchial artery; HP, hepatic portal vein; IOC, inner optic circle; ISV, intersomitic vessel; L, liver; LDA, lateral dorsal aorta; MsV, mesencephalic vein; NCA, nasal ciliary artery; OV, optic vein; P, pronephros; PAV, parachordal vessel; PCeV, posterior cerebral vein; PCV, posterior cardinal vein; PHBC, primordial hindbrain channel; PICA, primitive internal carotid artery; PMBC, primordial midbrain channel; PrA, prosencephalic artery; SB, swim bladder; SIA, supraintestinal artery; VA, ventral aorta; VTA, vertebral artery. Figure created with BioRender.com. **(B)** Timeline of key developmental milestones, organized by vascular system—Trunk, Brain, Fin, and Lymphatic—spanning 14 hpf to adulthood. In the trunk, vasculogenesis and arteriovenous identity establishment in the DA and PCV precede circulation onset (∼24 hpf), followed by intersegmental vessel (ISV) sprouting and DLAV formation, with gut and liver vascularization occurring later (∼5 dpf). Brain vascular development proceeds from cranial angioblast clustering and PHBC formation to establishment of brain circulation via the basilar and central arteries (BA/CtA), culminating in blood-brain barrier (BBB) maturation through progressive glial coverage. Fin vasculature begins with caudal fin vessel sprouting (∼48 hpf), followed by combined pectoral and caudal fin vascular remodeling that produces the mature three-vessel caudal fin pattern by 4 weeks post-fertilization (wpf); this vasculature retains regenerative capacity into adulthood. Lymphatic vessels arise from PCV-derived lymphatic sprouting, followed by the formation of parachordal lymphangioblasts and the maturation of the thoracic duct and lymphatic network. Red boxes in **(B)** indicate discrete, point-in-time developmental events (circulation onset; adult vascular regeneration) rather than extended processes.

Vascular development in zebrafish is comparable to that in humans, involving fundamental processes such as vasculogenesis, angiogenesis, and vascular remodeling, all of which are tightly regulated by molecular pathways (Figure 2). This section focuses on the development of trunk, brain, and fin vessels, which are among the most comprehensively studied vascular systems in zebrafish. Lymphatic vessel development is also considered. Detailed developmental timelines and vessel nomenclature across these vascular systems are summarized in Figure 2B.

### 2.1 Trunk vessels development

Vasculogenesis begins around 14 hpf, when mesodermal progenitors differentiate into endothelial cells to form the dorsal aorta (DA) and posterior cardinal vein (PCV) (Fouquet et al., 1997; Jin et al., 2005). Subsequent trunk and brain vessels arise through angiogenesis, followed by vascular remodeling that optimizes the network for efficient metabolite delivery (Isogai et al., 2001). The intersomitic vessels (ISVs), which sprout from the DA around 22 hpf, are among the earliest and most widely used models of angiogenesis in zebrafish, and cardiac contraction with circulating blood cells becomes detectable shortly after 24 hpf (Stainier et al., 1996).

Arterial and venous identity is acquired concurrently during primary vessel assembly (Gore et al., 2012; Roman et al., 2002). Although earlier models proposed that arteriovenous identity is determined by hemodynamic forces such as blood pressure (Gonzalez‐Crussi, 1971), molecular differences between arterial and venous precursors are now known to be established before the onset of flow, indicating that this identity is genetically imprinted rather than solely flow-dependent (Zhong et al., 2001). Uniquely among vertebrates, the zebrafish DA and PCV are single, unpaired trunk vessels (Isogai et al., 2001). Blood flow velocity increases progressively in the DA through larval development, while remaining comparatively lower and more stable in the PCV, reflecting early functional divergence between the arterial and venous circuits and establishing a physiologically relevant system for studying flow-dependent vascular mechanisms (Bagatto and Burggren, 2006; Watkins et al., 2012; Benslimane et al., 2020; Santoso et al., 2019; Subendran et al., 2021).

Trunk vessel formation is driven by coordinated tip and stalk endothelial cell behavior (Siekmann and Lawson, 2007). Tip cells extend filopodia and migrate in response to VEGF signaling, while the trailing, proliferative stalk cells elongate and stabilize the nascent sprout (Gerhardt et al., 2003; Jakobsson et al., 2010). Tip cell selection is governed by VEGFR2 expression: the highest-expressing cell upregulates Delta-like 4 (DLL4), activating Notch signaling in the adjacent cell to suppress its VEGFR2 expression and enforce a single tip cell per sprout, with Jagged-1 acting as an additional Notch modulator (Jakobsson et al., 2010; Hellstrom et al., 2007; Benedito et al., 2009). This VEGF-Notch crosstalk is essential for balanced angiogenic sprouting and vascular patterning throughout zebrafish development.

### 2.2 Brain vessels development

Brain vasculature develops independently of the trunk, arising from cranial angioblast clusters in the anterior mesoderm rather than the lateral plate mesoderm (Gupta et al., 2021). The rostral and midbrain organizing centers give rise to the earliest brain vessels, including the primordial hindbrain channels (PHBC), by 24 hpf (Breier et al., 1992; Quiñonez-Silvero et al., 2020). Flow initiates in the brain circulation shortly thereafter, and subsequent angiogenic sprouting from the PHBC establishes the basilar artery (BA) and central arteries (CtAs), which together form the first functional cerebral circulatory loop by around 48 hpf (Figure 2B) (Isogai et al., 2001; Quiñonez-Silvero et al., 2020; Chen et al., 2012; Isogai et al., 2001; Gore et al., 2012).

Brain vessel sprouting and patterning occur in proximity to developing neuronal clusters, suggesting bidirectional neurovascular communication as early as 36–48 hpf (Gupta et al., 2021). Glial-vascular interactions further support blood-brain barrier (BBB) maturation: glial coverage of brain vessels increases progressively through larval development alongside suppression of endothelial transcytosis, although full maturation occurs later than in mammals (Gall et al., 2025). This developmental sequence underscores zebrafish as a valuable model for angiogenesis, neurovascular development, and BBB formation.

### 2.3 Fin vessel development

Pectoral and caudal fin vasculature develop through distinct, well-staged processes that reflect their different roles in locomotion and regeneration, respectively (Figure 2B) (Paulissen et al., 2022; Yano et al., 2012; Oppenheimer and Chao, 1984; Huang et al., 2003; Leonard et al., 2023). Pectoral fin vascularization begins in late embryogenesis with sprouting from the common cardinal vein, followed by formation of the primitive pectoral artery and vein, and culminates over several weeks in a mature fin-ray vascular network connected to the dorsal aorta (Paulissen et al., 2022; Yano et al., 2012; Oppenheimer and Chao, 1984).

The caudal fin vasculature develops considerably faster and is a preferred model for studying vascular regeneration, owing to its segmented, easily visualized fin rays, each supplied by a single artery and paired veins (Huang et al., 2003; Hlushchuk et al., 2016). Vessels sprout from the DA and PCV extend into the fin fold beginning around 48 hpf, arborizing into a proximal plexus that progressively expands and remodels into a mature three-vessel pattern by 4 weeks post-fertilization (Paulissen et al., 2022; Oppenheimer and Chao, 1984; Leonard et al., 2023).

### 2.4 Lymphatic vessels development

Zebrafish lymphatic vessels form a distinct system that drains interstitial fluid, macromolecules, and immune cells, and have become a key model for lymphangiogenesis, building on the imaging and genetic advantages of the model described above (McKinney and Weinstein, 2008; Yaniv et al., 2006; Feng et al., 2021). As in mammals, lymphatic vessels arise from sprouting venous endothelial cells of the PCV beginning around 48 hpf, giving rise to the thoracic duct and the intersegmental and dorsal longitudinal lymphatic vessels (Figure 2) (Wilkinson and van Eeden, 2014).

Lymphatic endothelial cells subsequently aggregate along the horizontal myoseptum to form the parachordal lymphangioblasts, the scaffold for trunk lymphatic development, from which the thoracic duct and superficial and cardinal lymphatic networks progressively form and mature over the following days (Jung et al., 2017; Hu et al., 2024). This process is regulated by conserved molecular pathways, including VEGFC-VEGFR3 signaling and PROX1-mediated lymphatic endothelial cell specification (Jung et al., 2017; Hu et al., 2024).

## 3 Role of the vasculature in the process of zebrafish tissue regeneration

In mammals, tissue repair is highly efficient in the liver, lung epithelium, kidneys, and gut, but is severely limited in other tissues, including the central nervous system and appendages. Instead, mammals rely on wound healing to repair damaged tissue, which is often imperfect and can result in scarring or fibrosis, depending on the severity of the injury (Eming et al., 2014; Erickson and Echeverri, 2018). In contrast, the adult zebrafish exhibits remarkable regenerative abilities, fully restoring its vasculature and damaged tissues, including appendages, heart muscles, brain, spinal cord, and retina (Gemberling et al., 2013; Sehring and Weidinger, 2020). Crucially, for successful tissue regeneration, a functional vasculature that supports tissue repair is needed to provide oxygen, nutrients, and molecular signals necessary for cellular proliferation and differentiation. Therefore, the unique regenerative potential of zebrafish has attracted broad attention from researchers studying this process, which is challenging to study in mammals. The hope is that, given that humans and other mammals have limited regenerative capacity, the regeneration mechanisms identified in zebrafish could be reinitiated in mammals.

### 3.1 Heart regeneration

In adult mammals and amphibians, cardiac injury or damage is generally followed by scar formation, with almost negligible cardiomyocyte regeneration (Poss et al., 2002). This is because necrotic and apoptotic cardiomyocytes are being replaced by a permanent non-contractile fibrotic scar (Bahit et al., 2018; Gerber et al., 2016). In contrast, adult zebrafish can regenerate fully functional cardiac tissue after cardiac injury, primarily by generating new cardiomyocytes (CMs). This makes them a unique vertebrate model for studying the cellular and molecular mechanisms of heart regeneration (Simões et al., 2020; Wang J. et al., 2011). In the last 2 decades, zebrafish have contributed significantly to advancing the understanding of cardiac repair and regeneration (Goldman et al., 2017; Jopling et al., 2012). Different zebrafish heart injury models have been established and extensively utilized, including ventricular resection, cryoinjury, genetic ablation, hypoxia, explant culture, and laser-induced injury (Chablais et al., 2011; González-Rosa et al.; Schnabel et al., 2011; Oberpriller and Oberpriller, 1974). In all of these models, a standard observation is the prompt coronary revascularization, which is essential for facilitating and maintaining cardiac regeneration (Marín-Juez et al., 2016). Restoration of coronary vasculature via angiogenesis ensures adequate perfusion of the injured myocardium, thereby promoting cardiomyocyte proliferation and tissue remodeling (Deveza et al., 2012). Hence, given the limited therapeutic interventions for advanced heart conditions, promoting vascular regeneration could be used to develop promising therapeutic approaches for treating various cardiovascular diseases, such as ischemic stroke (Chen et al., 2019). Using a myosin light-chain 2a (mlc2a) enhanced GFP (EGFP) transgenic zebrafish line, Raya et al. showed that adult zebrafish have a unique ability to regenerate the heart. This regeneration includes upregulation of the msxB (Raya et al., 2003). A more recent study revealed that a transiently activated epicardial progenitor cell (aEPC) subpopulation, characterized by ptx3a and col12a1b expression, participates in cardiac tissue regeneration (Xia et al., 2022). The aEPCs originate from the epicardial epithelium, migrate to encompass the wound, undergo epithelial-mesenchymal transition (EMT), and develop into mural cells and pdgfra + hapln1a + mesenchymal epicardial cells following cardiac injury. Conditional ablation of aEPC impairs cardiac regeneration by restricting nrg1 expression and reducing mesenchymal cell numbers. These findings identify a transient adult epicardium progenitor population that is coordinating its role in myocardial and vascular regeneration (Xia et al., 2022). In addition, Wnt/β-catenin signaling is also necessary for cardiomyocyte proliferation and dedifferentiation, as well as scar regulation during regeneration (Bertozzi et al., 2022). Cardiomyocyte-specific conditional suppression of the pathway demonstrates that Wnt/β-catenin signaling promotes cardiomyocyte proliferation cell-autonomously (Bertozzi et al., 2022). These findings support a model where Wnt/β-catenin signaling promotes heart regeneration in zebrafish (Bertozzi et al., 2022). The established zebrafish models for heart regeneration have advanced understanding of the temporal molecular mechanisms underlying the regenerative response, including vascular remodeling and coronary vessel neogenesis (Bowley et al., 2021).

### 3.2 Fin regeneration

The regenerative capacity of zebrafish is most apparent in restoring fin vessels. The zebrafish caudal fin is characterized by a simple, thin architecture and relative transparency, making it an ideal tissue for studying vascular regeneration in adult zebrafish (Poss et al., 2003). The caudal fin amputation model has been widely used to study the mechanisms underlying fin vessel regeneration and restoration. While zebrafish larvae regenerate their fins in a few days, adult fish restore them within a couple of weeks after successive amputations (Azevedo et al., 2011). Further studies have shown that the regenerated vessels in zebrafish fins develop from vein-derived cells that acquire angiogenic potential and then migrate to form organized vessels in response to chemokine signaling (Xu et al., 2014; Chávez et al., 2016; Hasan and Siekmann, 2015). Also, the zebrafish caudal fin has been used to investigate the role of vascular endothelial growth factor receptor (VEGFR) signaling in regulating tail regeneration (Bayliss et al., 2006). Likewise, activating the neuronal voltage-gated sodium channel (VGSC) gene *scn8ab* promotes innervation of the appendage stump (Osorio-Méndez et al., 2022). In addition, blood vessel maturation is accompanied by the recruitment of peri-endothelial cells, known as mural cells, which form the contractile layers surrounding the vessels (Wilkinson and van Eeden, 2014). Current research focuses on the role of mural cells (including pericytes and smooth muscle cells) in fin vessel regeneration. Recent work has shown that regenerating vascular mural cells in zebrafish fin blood vessels do not arise from pre-existing mural cells. Instead, they arise from distinct progenitor populations that express fibroblast-specific genes and require Platelet-Derived Growth Factor Receptor Beta (PDGFRb) signaling (Leonard et al., 2022). PDGFRb plays a crucial role in the differentiation and function of these mural cells. Importantly, PDGFRb mutants and inhibition of PDGFRb signaling following injury severely impair mural cell recruitment and expansion, underscoring the critical role of this pathway in vascular homeostasis and regeneration (Leonard et al., 2022; Rajkumar et al., 2006). However, additional work is needed to fully understand the zebrafish model’s potential for vascular regeneration during fin regeneration.

### 3.3 Brain and spinal cord regeneration

Vascular development is very crucial for brain regeneration, particularly after ischemic insults like stroke or spinal cord injury (Chen et al., 2019). Restoration of the neural pathway involves the regeneration of neural cells and the development of an appropriate vascular structure to deliver essential nutrients and signaling molecules for optimal tissue repair. Adult zebrafish brain regeneration involves rapid and coordinated revascularization that supports neuronal survival, proliferation, and differentiation (Gemberling et al., 2013). Following brain injury in adult zebrafish, blood vessels rapidly remodel and sprout new vessels, thereby restoring the oxygen and nutrient supply that is critical for neurogenesis and tissue repair. Radial glial cells serve as neural progenitors that actively proliferate, supported by vascular niches that provide trophic and paracrine signals essential for regeneration (Gemberling et al., 2013; Alper and Dorsky, 2022). Further, the regeneration process is driven by endothelial cell proliferation and angiogenesis, guided by growth factors like VEGF and supported by interactions with glial cells and pericytes (Gupta et al., 2021; Kroehne et al., 2011). Molecular pathways, including VEGF, Notch, and hypoxia-inducible factors (HIFs), are pivotal in coordinating vascular and neuronal repair. Further, the BBB undergoes dynamic remodeling during regeneration to re-establish CNS homeostasis while allowing immune cell infiltration necessary for clearing damaged tissue (Noorimotlagh et al., 2017). Notably, zebrafish experience minimal scar formation, enabling efficient neurovascular coupling and brain function restoration (Chávez et al., 2016). Insights from zebrafish studies offer promising directions for developing therapies to enhance human vascular and neural repair.

Spinal cord regeneration in adult zebrafish is characterized by the formation of a glial bridge at the injury site over which regenerating axons traverse to reconnect with their targets (Saraswathy et al., 2024). Endothelial cells at the injury site proliferate and form new blood vessels that not only restore blood flow but also create a supportive microenvironment that promotes neural progenitor activity and axon regrowth. Key signaling pathways, such as Wnt/β-catenin, enhance neurogenesis, whereas fibroblast growth factor (FGF) signaling promotes glial proliferation and bridge formation (Saraswathy et al., 2024). Notably, adult zebrafish maintain neurogenic zones and sustain progenitor cell populations essential for CNS repair, in contrast to the limited neurogenesis in adult mammals (Chen et al., 2024). Immune cells dynamically modulate inflammation to favor regeneration rather than scarring, often in close interplay with vascular and glial components (Tendolkar and Mokalled, 2025). Accordingly, these insights into adult zebrafish brain and spinal cord regeneration hold promise for guiding therapeutic strategies to enhance CNS repair in mammals.

## 4 Zebrafish toolbox for studying vascular development

Building on the developmental features described above, zebrafish offer an extensive experimental toolbox for visualizing and genetically manipulating the vasculature *in vivo* (Gore et al., 2012; McKinney and Weinstein, 2008). In this section, we discuss imaging techniques for visualizing zebrafish vasculature, as well as genetic tools applicable to zebrafish vascular research.

## 5 Imaging techniques of zebrafish vasculature

Multiple *in vitro* and *in vivo* assays were designed to investigate the cellular and molecular mechanisms underlying vasculogenesis and angiogenesis (Cimpean et al., 2011). Integrating these assays is essential for understanding vasculature development. *In vitro*, specific behaviors of endothelial cells, including proliferation, differentiation, migration, and tube formation, are induced by external stimulatory or inhibitory agents. These *in vitro* systems enable the identification of molecular mechanisms underlying angiogenesis (Irvin et al., 2014). Such assays are common practice as they are quantitative, reproducible, easily monitored and implemented, and provide the confidence required for screening potential pro- or anti-angiogenic compounds (Weiss et al., 2015). Additionally*, in vivo* assays are important for studying interactions among multiple cell types during angiogenesis and vasculogenesis and for understanding the translational relevance of results obtained from *in vitro* assays (Chávez et al., 2016). Notably, *in vivo* tests offer a three-dimensional (3D) environment for cell-cell interactions (Morrissey and Sherwood, 2015). This is attributable to the presence of basement membranes, which underlie epithelia and endothelia. Basement membranes were traditionally assumed to be a static support structure. However, an increasing body of evidence demonstrates that basement membrane deposition is dynamic and alterations in the basement membrane induce cellular responses, which can mechanically sculpt different tissues (Morrissey and Sherwood, 2015).

Because of the transparency of the embryonic zebrafish, a wide array of techniques has been developed for high-resolution imaging, tracking, and functional manipulation of its vasculature. These techniques help explore ubiquitous events in vasculogenesis and angiogenesis (Vascotto et al., 1997). In addition to techniques for assessing embryonic vascular development, methods for assessing adult vascular development are available (Kamei et al., 2010). Classical studies relied on light microscopy to identify blood and endothelial progenitor cells (Sabin, 1920). Blood vessels can be labeled using several methods, including *in situ* hybridization (RNA-based), immunohistochemistry (IHC) (protein-based) with vascular-specific antibodies, resin injection, and alkaline phosphatase staining. These methods enable static visualization of the vasculature because the zebrafish specimens are fixed during processing. To overcome the static nature of vessel imaging, approaches such as microangiography and vascular-specific transgenic fluorescent fish lines have been developed as alternative methods for visualizing vessels in live embryos (Cha and Weinstein, 2007). Table 1 summarizes the techniques used to image and assess zebrafish vasculature in developing embryos and adults.

**TABLE 1:** Techniques used for assessing vascular development in zebrafish.

Assay type	Use	Assay	Setting	Advantages	Disadvantages	References
Microscopy	*In vivo* imaging	Light microscopy	*In vivo* imaging of zebrafish vasculogenesis	Provides detailed morphogenetic information on the cellular and subcellular levels, non-invasive	Limitations in optical penetration depth	Keller (2013), Lawson and Weinstein (2002a)
	*In vivo* imaging of vascular function	Fluorescent microscopy	Real-time visualization and quantification of blood flow, 3D imaging of endothelial barrier function	High-resolution and imaging speed combine to measure physiologic function with molecular behavior	Lack of optical sectioning capability and unsuitable for 3D imaging	Watkins et al. (2012)
	3D spatial mapping of vasculature	Light-sheet fluorescence microscopy (LSFM)	Imaging vascular development during embryogenesis	Faster, reduced photodamage, and offers optical sectioning	Often requires modifications in the experimental design and has illumination issues	Abu-Siniyeh and Al-Zyoud (2020), Park et al. (2015)
	Provides an ultrastructural context in a fixed cell	Correlative volume electron microscopy	Imaging transient blood vessel fusion events	High resolution can map biological events in relation to the surrounding tissue environment	Requires ultrathin sections, which could introduce artifacts and damage specimens	Armer et al. (2009)
	​	Correlative light and electron microscopy	Imaging vascular morphogenesis in living zebrafish embryos and correlating it with electron micrographs and tomograms	Gives an exact correlation between light and electron microscopy images	Technically challenging, expensive	Goetz et al. (2015)
	*In vivo* imaging of microvasculature	Two-photon fluorescence microscopy	3D reconstruction of the microvascular networks	High resolution can image optically thick specimens, with less scattering, and eliminates photobleaching of fluorescent molecules	The limitation of optical range and scanning speed produces considerable photodamage	Abu-Siniyeh and Al-Zyoud (2020), Zeng et al. (2014)
	*In vivo* imaging of microvasculature	Multiphoton fluorescence microscopy	Imaging angiogenic network formation in living *Tg(fli1: EGFP)* ^*y1*^ zebrafish embryos	Higher efficiency and yield, less scattering, allow imaging from structures deep within a tissue block, lower phototoxicity	Photobleaching and photodamage	Isogai et al. (2003)
	Imaging complex *in vivo* developmental processes in zebrafish	Second harmonic generation	Study collagen organization during wound healing, study gene expression in the vasculature	Direct visualization of the tissue structure, high resolution, less photobleaching and phototoxicity than fluorescence methods	Limited depth of penetration, and often not adequate for *in vivo* applications	LeBert et al. (2015), LeBert et al. (2016), Hsieh et al. (2008)
​	Studying the vascular dynamics	Structured illumination microscopy (SIM)	Imaging the dynamics of the cardiovascular system and cerebral vessels	Fast imaging capability, high throughput, and reduced complexity	Lower image quality	Chong et al. (2020)
Molecular	*In vivo* imaging	Confocal microangiography	Assessing blood vessels in active circulation and lymphatic vessels	Enables detailed study of the vessels’ pattern, function, luminal space, and integrity. Easy to perform	It cannot be used for studying vessels lacking circulation, is not suitable during embryogenesis, and in the late stages	Jung et al. (2016)
	Imaging gene expressions within blood vessels	*In situ* hybridization	Assessing the spatial and temporal patterns of vascular genes	Helps determine the relation between the distribution of specific nucleic acids and protein products	Difficulty in identifying targets that have low DNA and RNA copies	Wang et al. (2020)
		Immunohistochemistry	Analyzing the lymphatic system in zebrafish using lymphatic endothelial markers	Allows studying the spatial and temporal protein expression	It is not standardized worldwide, requires costly equipment, and is subject to human error	Shimoda and Isogai (2012)
	Non-vital imaging of blood and lymphatic vessels	Micro-dye injection	Visualizing the developing circulatory system	Detailed visualization of the luminzed vessels in embryos	Not suitable for adult zebrafish	Kamei et al. (2010)
		Micro-resin injection	Visualizing larger adult blood vessels, such as the renal vasculature	It could be used to visualize vessels at almost any stage of development	Technically challenging, especially on small specimens	Kamei et al. (2010), Jung et al. (2016)
		Alkaline phosphatase (AP) staining	Assessing endogenous AP activity in zebrafish xenograft angiogenesis	Easy and rapid visualization of the vasculature in many specimens	Lower resolution than other methods	Nicoli and Presta (2007), Nicoli et al. (2007)
	Analyzing gene function	Microinjection	Delivering solutions containing genetic material or knockdown constructs into the blastomeres	Rapid and efficient technique, allowing the injection of hundreds of embryos per hour	Requires specialized equipment and technical skills to prevent cell damage	Rosen et al. (2009), Cianciolo Cosentino et al. (2010)
	Vital imaging of blood and lymphatic vessels	vascular-specific transgenic zebrafish	Genetic screening for identifying vascular-defected mutants	Visualizing vessels that are not functional	​	Cha and Weinstein (2007)
Computational analysis	Automated 3D vascular quantification	ZVQ (Zebrafish Vascular Quantification)	Segmentation, inter-sample registration, and quantification of 3D cerebrovascular architecture from light-sheet fluorescence microscopy datasets	Open-source; extracts volume, surface area, density, branching points, length, radius, and network complexity; enables inter-sample registration	Optimized for cerebral vasculature; requires light-sheet fluorescence microscopy datasets	Kugler et al. (2022)
​	Automated segmentation, skeletonization, and topological quantification	VISTA-Z pipeline	Standardized vascular quantification across diverse confocal fluorescent datasets and developmental stages	Fully automated, Python-based workflow; adaptive contrast enhancement; skeleton-based topology analysis with branchpoint refinement; validated across multiple transgenic lines and mutant phenotypes	Relies on 2D maximum-intensity projections rather than full 3D reconstruction; requires computational/coding familiarity	Rodriguez-Pastrana et al. (2026)

### 5.1 Microscopy techniques

Multiple microscopy techniques have been used and developed for imaging vascular tissues in zebrafish embryos and larvae. Standard light microscopy is a commonly used technique for vascular imaging, particularly in transparent embryos. This technique can provide detailed morphology information on the cellular and subcellular levels in a non-invasive manner (Keller, 2013). However, this technique has certain limitations, including limited optical penetration depth during live imaging of zebrafish embryos. Such limitations have been overcome through the integration of advanced microscopy techniques, including fluorescence correlation spectroscopy (FCS), Multiphoton microscopy (MPM), light-sheet microscopy (LSM), and second-harmonic generation (Table 1). These imaging methods have facilitated the discovery of internal organ functions in zebrafish and helped understand pathophysiological events (Chávez et al., 2016). These advanced imaging techniques can also run at high speed over extended time points, enabling dynamic, whole-embryo visualization of morphological processes as they form in both normal and mutant embryos. This has contributed to comprehensive zebrafish gene expression atlases with high spatiotemporal resolution. Using these images, entire organs and tissues in the zebrafish model have been reconstructed, revealing the dynamic building plans of a developing embryo. In addition, defective processes in mutant embryos were revealed.

### 5.2 Molecular techniques

Non-vital blood vessel imaging techniques primarily rely on intravascular injection of dyes or fluorescent compounds to visualize the vasculature in fixed zebrafish specimens. Some of the widely used injection methods for non-live imaging include micro-dye injection and micro-resin injection, which rely on injecting dyes or plastic resins into lumenized or opened blood vessels connected to the systemic circulation (Kamei et al., 2010). The selection of the appropriate method depends on the stage of zebrafish development. Dye injections are often used for embryos and larvae, whereas resin injections are used for adults (Jung et al., 2016). While these methods are mainly used for visualizing the vasculature in non-living zebrafish specimens, zebrafish are mostly known for their convenience for live imaging techniques. In this regard, several strategies and methods were developed for assessing blood vessels in active circulation and lymphatic vessels. One such approach is confocal microangiography (Jung et al., 2016), which has been used for visualizing patent blood vessels in zebrafish (Isogai et al., 2001; Weinstein et al., 1995). These methods have also been utilized to comprehensively describe the developing vasculature anatomy in zebrafish through a combination of microangiography with fluorescent microspheres and confocal laser microscopy (Isogai et al., 2001; Weinstein et al., 1995). Using this method, fluorescent microbeads are injected into the circulatory system of living zebrafish embryos to construct detailed 3D images of the vascular anatomy (Weinstein et al., 1995). Although microangiography is considered the tool of choice for imaging vessels in living embryos, it has significant limitations. Since microangiography can label only the lumens of assembled, functional vessels, it cannot visualize the dynamic process of vessel formation during embryogenesis. Also, this method is unsuitable for use during later stages of zebrafish development, including larvae, juveniles, and adults. Alternatively, classical intravascular injection methods are considered to be more suitable for visualization of the blood or lymphatic vessels during these later stages of zebrafish development (Yaniv et al., 2006). Other commonly used molecular techniques for vascular imaging are summarized in Table 1.

### 5.3 Imaging vasculature in transgenic zebrafish lines

A significant advantage of the zebrafish model is its transparency. This has been exploited using tissue-specific vascular gene promoters, now readily available, that drive fluorescent reporter proteins in a range of colors (Simons et al., 2015). Thus, vascular patterning research has gained attention following the generation of numerous transgenic zebrafish lines that express specific fluorescent proteins in vascular endothelial cells (Jin et al., 2005; Choi et al., 2007; Lawson and Weinstein, 2002). Establishing these transgenic fluorescent lines has facilitated high-resolution and high-speed *in vivo* imaging and assessment studies for the developing vasculature and the dynamics of vessel growth and remodeling (Cha and Weinstein, 2007). Also, unlike micro-dye or resin injection techniques, transgenic zebrafish lines offer the advantage of repeated long-term dynamic visualization of endothelial cell behavior in living animals, especially when multiphoton imaging is employed (Cha and Weinstein, 2007). Zebrafish transgenic lines are mainly used for genetic and chemical screening and experimental manipulations to advance the knowledge regarding assembling complex vascular networks (Cha and Weinstein, 2007). Further, these lines allow studying the morphological developmental dynamics of vessel formations at the sub-cellular level using confocal microscopy (Simons et al., 2015). The zebrafish toolbox has been further expanded by reporter lines that distinguish endothelial subtypes lining the vessels—arterial, venous, and lymphatic—using flow cytometry or fluorescence-activated cell sorting. These techniques can also localize endothelial subcellular structures as cytoplasmic, nuclear, or membrane-restricted (Lawson and Weinstein, 2002).

The generation of zebrafish transgenic lines has provided a unique opportunity to expand current knowledge of vasculogenesis. However, these animals are not well-suited for assessing vascular function, as it is challenging to visualize specific vessels (Schmitt et al., 2012). Conversely, these fluorescent lines are well-suited for visualizing nonfunctional vessels during assembly, or the individual migration of endothelial cells or angioblasts that have not yet been incorporated into the vascular cord. Alternatively, tracking fluorescently labeled or non-labeled circulating red blood cells (RBCs) using bright-field or fluorescent microscopy equipped with a high-frame-rate camera could provide such information (Schmitt et al., 2012). Moreover, transgenic lines can be used for functional testing at the level of vessels, including assessment of perfusion using the double-transgenic mutant Tg (kdrl:GFP; gata1:DsRed) to monitor circulating erythrocytes (Traver et al., 2003). Fluorescent proteins, such as green fluorescent protein (GFP), have been used to label various tissues in transgenic zebrafish embryos and larvae (Jung et al., 2016). Fli:EGFP transgenic line is one of the most widely used lines for studying vasculogenesis due to its specificity and brightness (Cha and Weinstein, 2007).

Several techniques allowing precise control of gene expression have been successfully adapted to the zebrafish model. One such approach is the binary Gal4/UAS (Upstream Activating Sequence) system, widely used in *Drosophila melanogaster* (Brand and Perrimon, 1993). In zebrafish, this system has been used to selectively express genes and to create tissue-specific expression of fluorescent proteins for generating transgenic lines. This technique offers the capacity to have precise temporal and spatial control of gene expression with the combination of Gal4 transcription factor and UAS sequences (Asakawa and Kawakami, 2008). In zebrafish, conditional and stable gene expression (Scheer and Campos-Ortega, 1999; Choe et al., 2012) has been accomplished using this method. This method allows researchers to investigate developmental processes and cell behaviors in vertebrate animal models (Li et al., 2025). In zebrafish, it was also used for the manipulation of primordial germ cells and enhanced fluorescent protein expression for visualization (Xiong et al., 2013). These studies demonstrate the versatility of the Gal4/UAS system for providing deep insights into gene function. In addition, the Gal4/UAS system has been used to study gene dosage effects and neurodegeneration in *Drosophila*. This shows that the system can facilitate investigations into biological responses to transcription factor interactions (Rezával et al., 2007). Table 2 summarizes the commonly used vessel-specific transgenic lines.

**TABLE 2:** Commonly used vessel-specific transgenic zebrafish lines.

Line	Marker genes	Vascular expression	Phenotype	Used to study	Illustration	References
Tg (5xUAS:cdh5-EGFP)	Cadherin 5 (CDH5)	Pan-endothelial	Normal blood vessel endothelial cell and cell-cell junction morphology Characterized by the presence of green fluorescence protein (GFP) in the vascular endothelial cells	Vascular endothelial cell Imaging, vascular development, vascular integrity and barrier function, drug screening, disease models related to vascular disorders, including studies of vascular diseases such as hemorrhage, edema, inflammation, periapical granuloma, and epithelioid Sarcoma	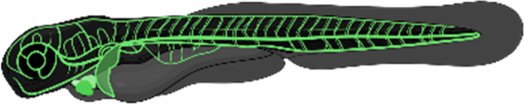	Sauteur et al. (2014), Stelzer G et al. (2023), Lenard et al. (2013), Hübner et al. (2018), Aydogan et al. (2015), Hultin et al. (2014), Bradford et al. (2022)
Tg (-7.8gata4:GFP)ae3	GATA Binding Protein 4 (GATA-4) transcription factor	Endocardial and myocardial cells	Normal blood vessel endothelial cell and cell-cell junction morphology Characterized by the presence of GFP in the developing heart tissue	Cardiac development, cardiac gene expression, and heart regeneration	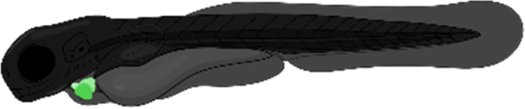	Astone et al. (2015), Heicklen-Klein and Evans (2004)
Tg (fli:eGFP)y1	Friend leukemia integration 1 (Fli-1) transcription factor	The cytoplasm of endothelial cells	Segmental vessels display ectopic sprouting and normal cell-cell junction morphology Characterized by the presence of enhanced GFP in the blood vessels and endothelial cells	Angiogenesis, tumor angiogenesis, endothelial cell behavior, and vascular diseases like atherosclerosis	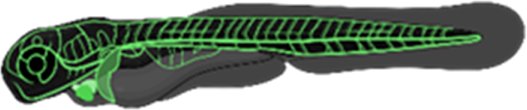	Lawson and Weinstein (2002), Delov et al. (2014)
Tg (fli1:neGFP)y7	Friend leukemia integration 1 (Fli-1) transcription factor	Nucleus of endothelial cells	Abnormal increase in the basal communicating artery blood vessel endothelial cells and posterior communicating artery blood vessel endothelial cells. Characterized by the presence of non-enhanced green fluorescence protein (neGFP) in the blood vessels and endothelial cells	Angiogenesis, tumor angiogenesis, endothelial cell behavior, and vascular diseases such as atherosclerosis	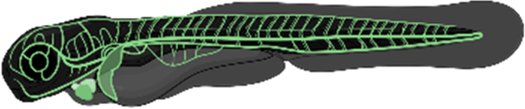	Siekmann and Lawson (2007), Wang et al. (2011c), Roman et al. (2002)
Tg (kdrl:eGFP)s843	kinase insert domain receptor-like (Kdrl) gene	Initially pan-endothelial, enriched in arteries at later stages	Abnormal increase in trunk vasculature artery hey2 expression, trunk vasculature artery notch1b expression, and trunk vasculature artery efnb2a expression. Characterized by the presence of enhanced green fluorescence protein (eGFP) in the vascular endothelial cells	Angiogenesis, tumor angiogenesis, endothelial cell behavior, and vascular diseases such as atherosclerosis	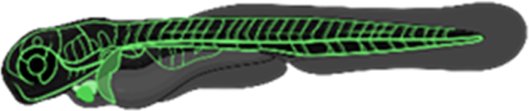	Ji et al. (2005), Chiavacci et al. (2015), Zhou et al. (2015)
Tg (kdrl:HsHRAS-mCherry)s896	kinase insert domain receptor-like (Kdrl) gene	Endothelial cells	Normal vasculature development and abnormal AV myocardial and endocardial cells’ cellular shapes Characterized by the presence of red fluorescence protein (mCherry) in the vascular endothelial cells, particularly in vascular development and angiogenesis areas	Vascular system and angiogenesis, such as atrioventricular canal formation	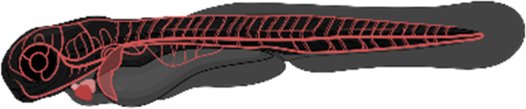	Schmid et al. (2013), Chi et al. (2008)
Tg (dll4:eGFP)	Notch ligand	Endothelial cells	Angiogenesis decreased occurrence, abnormal Characterized by the presence of enhanced green fluorescence protein (eGFP) in vascular endothelial cells, specifically in areas of active angiogenesis and vascular development	Vascular development, with a particular focus on the role of dll4 in angiogenesis and Notch signaling	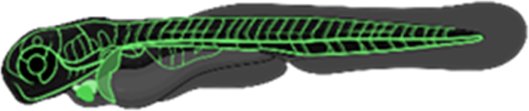	Bradford et al. (2022), Covassin et al. (2006)
Tg (flt4:YFP)	fms-related receptor tyrosine kinase 4 (flt4) or Vegfr3	Early on pan pan-endothelial and later restricted to the vein only	Deficient in lymphatic vascular development Characterized by the presence of yellow fluorescence protein (YFP) in lymphatic endothelial cells, mainly in areas related to lymphatic vessel development	Lymphatic vessel development and the expression of genes associated with lymph formation	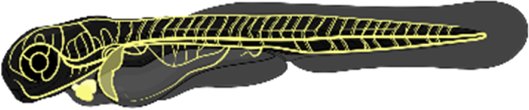	Hogan et al. (2009)
Tg (gata1a:DsRed)	GATA-1transcription factor	Erythroid progenitor cell	Characterized by the presence of red fluorescence protein (DsRed) in red blood cells and cells involved in hematopoiesis	Hematopoiesis and erythropoiesis	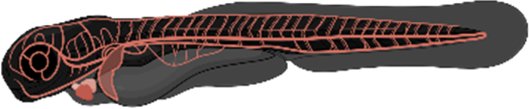	Yaqoob et al. (2009)

### 5.4 Computational and automated quantification of vascular networks

While transgenic fluorescent reporter lines and advanced microscopy enable detailed visualization of the zebrafish vasculature, extracting reproducible, quantitative data from these images has historically relied on manual measurement, which is time-consuming, subjective, and difficult to scale across large datasets. To address this, automated image analysis pipelines have been developed to segment, skeletonize, and topologically quantify the zebrafish vascular network. Zebrafish Vascular Quantification (ZVQ) provides an open-source 3D image analysis workflow for the zebrafish cerebrovasculature, combining inter-sample registration with extraction of quantitative parameters such as vessel volume, surface area, density, branching points, length, radius, and network complexity (Kugler et al., 2022). More recently, the VISTA-Z pipeline was developed as an automated, Python-based tool that standardizes vascular quantification across diverse fluorescent transgenic datasets, combining adaptive contrast enhancement, vessel segmentation, and skeleton-based topology analysis with refined branchpoint detection; it has been validated for detecting both vessel loss and hyper-angiogenic phenotypes in zebrafish mutants (Rodriguez-Pastrana et al., 2026). These computational pipelines bridge image acquisition and disease-modelling applications by enabling reproducible, high-throughput phenotyping of vascular structure, complementing the transgenic and imaging tools described above and facilitating quantitative comparisons across genotypes, developmental stages, and pharmacological treatments. A summary of these computational tools is included in Table 1.

## 6 Genetic tools in zebrafish

Unlike mice or humans, teleost species, including zebrafish, have gene duplication in approximately 20% of genes (Howe et al., 2013), which makes it challenging to assign functions to zebrafish genes. Nevertheless, approximately 70% of human genes have at least one orthologue in Zebrafish, and approximately 84% of human disease-causing genes have a zebrafish counterpart (Howe et al., 2013). Thus, zebrafish offers a genetic manipulation system that can probe mechanisms underlying human diseases (Sehring and Weidinger, 2020) affected by the vasculature. Also, zebrafish embryos can survive early development without functional vasculature, allowing the study of vascular mutations that otherwise cause embryonic death in knockout mouse models. Zebrafish are also amenable to both forward and reverse genetic analysis and techniques, providing opportunities for genetic screens and identifying mutations in novel genes regulating vascular development (Gore et al., 2012). For example, large-scale mutagenesis screening has identified several mutant lines that are suitable for studying aberrant cardiac and vascular development (Stainier et al., 1996; Chen et al., 1996). With the completion of the zebrafish genome sequencing project, it has become possible to conduct a systematic analysis of the function of zebrafish genes (Varshney et al., 2013).

Other genetic techniques that have been used in zebrafish research include gene knockdown by morpholino-modified oligonucleotides, transposon-mediated transgenesis, gene knock-in, and knockout technologies (Lawson and Wolfe, 2011; Hwang et al., 2013). Gene knock-in and knock-down editing tools, including transcription activator-like effector nucleases (TALENs), zinc-finger nucleases (ZFNs), and CRISPR/Cas9, have all been applied to the zebrafish model for editing specific genes associated with the zebrafish vasculature (Asnani and Peterson, 2014; Simons et al., 2015; Hwang et al., 2013). Gene knockout is also achievable in zebrafish using CRISPR/Cas9 and other targeted mutagenesis approaches, enabling precise disruption of gene function for vascular research (Shin et al., 2023). These modern approaches have complemented conventional gene knockdown approaches, such as morpholino antisense oligonucleotides. Introducing mutations in a zebrafish gene can cause the protein to lose its native activity (loss-of-function, LOF approach) or confer a new function (gain-of-function, GOF approach). These strategies are discussed next in relation to vascular development.

### 6.1 Loss-of-function (LOF) approach in zebrafish

The LOF approach involves disrupting gene activity to study the resulting phenotypes and elucidate gene function. In zebrafish, this can be achieved using multiple genetic tools, including extensive availability of mutant strains, targeted mutagenesis with synthetic morpholinos, small-molecule inhibitors, and advanced conditional gene manipulation systems such as Cre-lox recombination (Housden et al., 2017). Morpholinos provide transient gene knockdown by blocking mRNA translation or splicing, though their effects diminish by 3 days post-injection. Because morpholinos are typically injected at the 1–2 cell stage, this approach is primarily effective up to ∼3 dpf, thereby limiting analyses to early developmental stages. In addition, CRISPR/Cas9 technology can also be used in zebrafish to rapidly screen for LOF phenotypes. For example, transient CRISPR-mediated mutants, known as crispants, can recapitulate known LOF phenotypes and therefore reduce both the time and cost associated with deep phenotyping of genes and regulatory elements. However, crispants do not replace the need for stable germline mutants for in-depth functional analyses due to mosaicism and variability in editing efficiency (Shah et al., 2015; Burger et al., 2016). A key challenge in LOF is gene redundancy, as knocking down a single gene may not produce an apparent phenotype (Zimmer et al., 2019). An important factor when studying gene function is that almost 20% of zebrafish genes are duplicated. Blocking one gene may not result in loss of function (LOF), since the duplicate gene may still function (Postlethwait et al., 2000). Additionally, concerns about off-target effects in RNA interference (RNAi) and CRISPR/Cas9 gene-editing approaches necessitate cautious interpretation of results. In the case of morpholinos, it is not possible to evaluate later developmental stages because morpholino efficacy is lost 3 days post-injection. Also, morpholinos can disrupt normal development if the targeted protein plays a ubiquitous role (Zimmer et al., 2019). Overall, combining these complementary LOF strategies, while accounting for their respective strengths and limitations, enables a comprehensive dissection of gene function in zebrafish vascular development and beyond.

### 6.2 Gain-of-function approach in zebrafish

The GOF approach is considered complementary to the LOF screening and is achieved by overexpressing genes in a tissue or cell-type-specific manner to determine their phenotypic consequences (Maddison et al., 2009). GOF can be achieved through transient mRNA injection or, more commonly, by generating stable transgenic zebrafish lines in which the gene of interest is integrated and expressed under specific promoters (Maddison et al., 2009; Sassen and Köster, 2015). These transgenic approaches facilitate large-scale genetic screens and the production of diverse lines for *in vivo* studies of gene function. Furthermore, overexpression of specific human disease-associated genes in zebrafish can lead to phenotypes resembling those of the human condition (Santoriello and Zon, 2012). This provides powerful models for investigating pathogenesis, identifying therapeutic targets, and screening drug candidates. For example, enforced expression of multiple domains of gicerin/CD146 (cell adhesion molecule) constructs resulted in deficits in angiogenic sprouting, most notably in the intersegmental arteries (So et al., 2010).

Limitations of GOF include mosaic expression patterns in transient assays, in which not all cells express the transgene consistently, which can complicate data interpretation (Sassen and Köster, 2015). Moreover, overexpression can induce cellular stress or toxicity, which may confound phenotypic analysis (Perkins et al., 2010). Therefore, it is critical to use multiple orthogonal methods—including stable transgenics, inducible systems, and complementary LOF approaches—to rigorously evaluate gene function in zebrafish.

## 7 Vascular disease modeling in zebrafish

**FIGURE 3:**
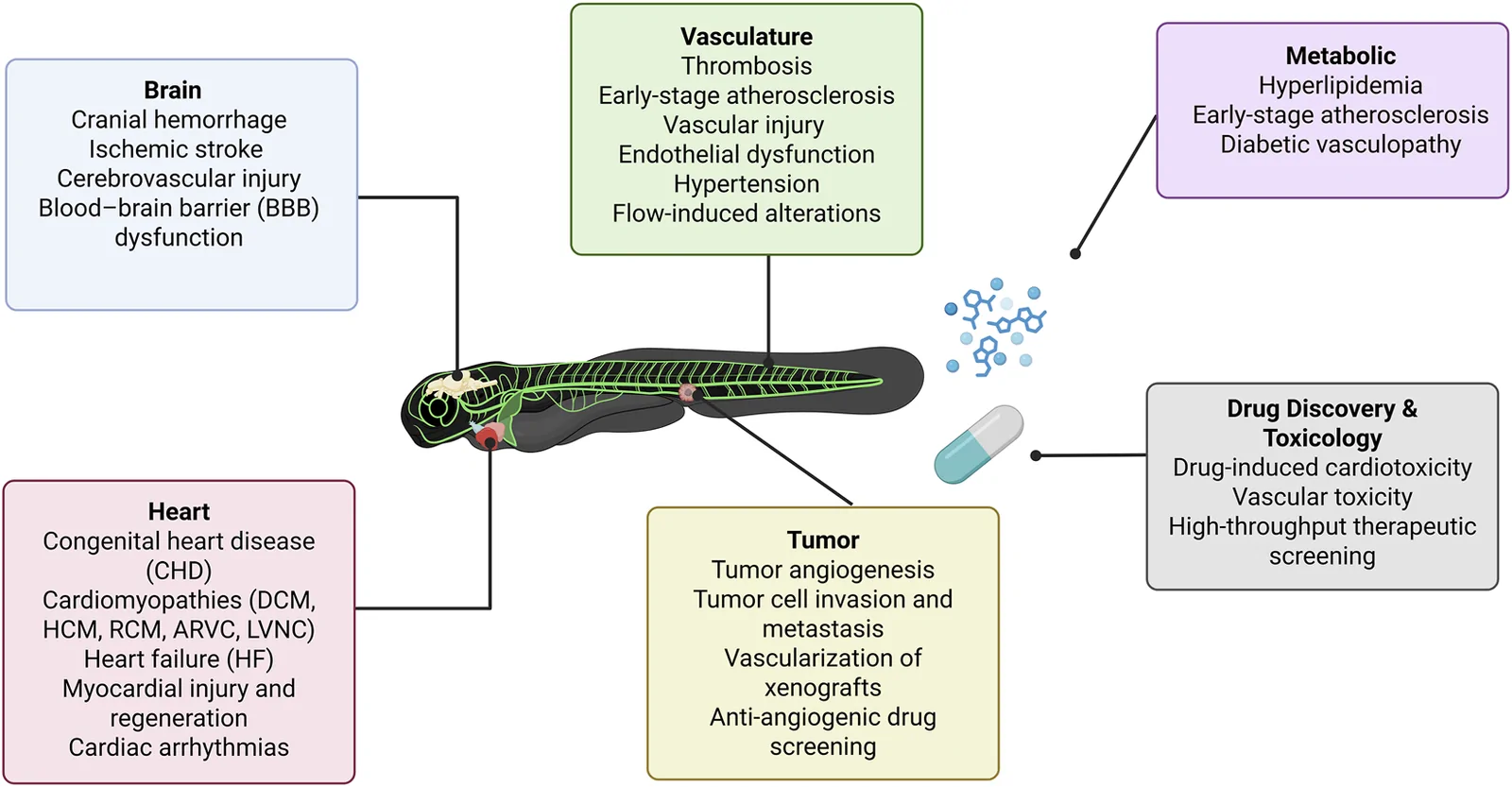
Zebrafish models for studying vascular and cardiovascular diseases. Zebrafish provide a versatile platform for investigating a wide range of vascular and cardiovascular disorders. These include cerebrovascular diseases, such as cranial hemorrhage, ischemic stroke, cerebrovascular injury, and blood–brain barrier dysfunction; cardiovascular disorders, including congenital heart disease, cardiomyopathies, heart failure, myocardial injury and regeneration, and cardiac arrhythmias; vascular pathologies, such as thrombosis, endothelial dysfunction, vascular injury, hypertension, and flow-dependent vascular alterations; and metabolic vascular complications, including hyperlipidemia, early-stage atherosclerosis, and diabetic vasculopathy. Zebrafish are also widely used to study tumor angiogenesis, cancer metastasis, drug-induced cardiovascular toxicity, and therapeutic screening. Collectively, these models offer valuable insights into disease mechanisms, facilitate target discovery, and support the development of novel vascular and cardiovascular therapies.

Zebrafish have been used to model a broad spectrum of vascular and cardiovascular diseases, spanning cerebrovascular, cardiovascular, vascular, and metabolic-vascular conditions, summarized schematically in Figure 3. Table 3 summarizes key zebrafish transgenic and mutant lines used to model specific human cardiovascular and vascular diseases, together with their key phenotypes and experimental applications.

**TABLE 3:** Zebrafish transgenic and genetic models for the study of human cardiovascular and vascular diseases.

Transgenic line	Mutated gene	Human disease modeled	Key phenotype	Purpose	References
Tg (mlc2a:EGFP)	Transgenic line expressing EGFP under the control of the wild-type myosin light chain 2a (mlc2a) promoter	Heart regeneration	EGFP expression in the myocardium	Visualization of cardiac development and heart morphology	Raya et al. (2003)
scn8ab mutant (Nav1.6-deficient)	Sodium voltage-gated channel alpha subunit 8b (scn8ab)	Regenerative biology model	Loss of scn8ab resulted in a reduction of fin innervation, impaired blastema formation, and defective fin regeneration following amputation	To determine the role of voltage-gated sodium channel Scn8a in nerve-dependent fin regeneration and tissue repair	Osorio-Méndez et al. (2022)
pdgfrb^um148^	platelet-derived growth factor receptor beta (pdgfrb)	Pericyte dysfunction and vascular repair disorders	Impaired mural cell recruitment and vascular regeneration	Assess the role of PDGFRβ signaling in vascular mural cell development and regeneration	Leonard et al. (2022)
Tg (fli1:EGFP) + cd146 morpholino	CD146/gicerin (mcam) knockdown	Tumor angiogenesis and vascular disorders	Defective ISV formation and impaired angiogenesis	Defective ISV formation and impaired angiogenesis	So et al. (2010b)
Tg (fli1:EGFP) + egfl7 morpholino	epidermal growth factor-like domain-containing protein 7 (egfl7) Knockdown	Angiogenesis defects, vascular malformations, and endothelial dysfunction	Defective vascular tubulogenesis, abnormal intersegmental vessels (ISVs), impaired angiogenic sprouting, and disrupted lumen formation	To investigate the role of Egfl7 in vascular development and angiogenesis	Nichol and Stuhlmann (2012)
Violet Beauregarde (vbg)	vbg encodes activin receptor-like kinase 1 (Acvrl1)	Vascular malformations include arteriovenous malformations (AVMs), such as hereditary hemorrhagic telangiectasia (HHT), an autosomal-dominant disorder caused by mutations in the Transforming Growth factor-β (TGF-β) signaling pathway	Enlarged cranial vessels and increased endothelial cell proliferation	Investigate the role of ALK1/TGF-β-BMP signaling in vascular development and vascular malformation formation	Roman et al. (2002)
rasa1a^ca35^ rasa1b^ca59^	rasa1a rasa1b	Capillary Malformation–Arteriovenous Malformation (CM-AVM) syndrome	Cavernous AVMs, abnormal blood flow, and reduced klf2a expression	Investigate the role of RASA1 in vascular development and AVM formation	Greysson-Wong et al. (2023)
Tg (kdrl:GFP)^la116^ + AGGF1 knockout	Angiogenic factor with G-patch and FHA domains 1 (AGGF1)	Klippel–Trenaunay syndrome (KTS), vascular malformations, abnormal angiogenesis	Impaired angiogenesis, defective vein differentiation, and abnormal vascular development	Investigate the role of AGGF1 in vein specification and angiogenesis during embryonic vascular development	Chen et al. (2013)
Santa (San, the zebrafish orthologue of KRIT1) Valentine (vtn, the zebrafish orthologue of CCM2) Heart of glass (heg)	krit1 (CCM1) CCM2 heg1	Cerebral cavernous malformations (CCMs)	Dilated heart, thin-walled non-patent vessels, defective lumen formation, and abnormal vascular morphogenesis	Investigate the CCM-related zebrafish mutant	Chan et al. (2010)
vhl^hu2117^ vhl^hu2081^	vhl −/−	von Hippel–Lindau (VHL)	Excessive VEGF-dependent angiogenesis, retinal vascular leakage, retinal detachment, brain and ocular neovascularization	Investigate hypoxia-driven angiogenesis and evaluate anti-angiogenic therapies	va et al. (2010)
Wild-type AB zebrafish + with high-cholesterol diet (HCD)	Cholesteryl ester transfer protein (cetp)	Hypercholesterolemia, dyslipidemia, atherosclerosis	Rapid diet-induced hypercholesterolemia and lipid accumulation	Study lipoprotein metabolism and early atherosclerotic processes	Kim et al. (2011)
AB strain + Apolipoprotein C-II (APOC2) loss of function	APOC2	Hyperlipidemia	Severe hypertriglyceridemia	Investigate the role of APOC2 in triglyceride metabolism and model hyperlipidemia-associated vascular disease	Liu et al. (2015)
AB strain + Idlr knockout	low-density lipoprotein receptor (Idlr)	Hyperlipidemia	Severe hypertriglyceridemia	Investigate LDL receptor function	Liu et al. (2018)
Tg (fli1a: EGFP) y	Friend leukemia integration 1a (fli 1a)	Tumor angiogenesis, vascular malformations, endothelial dysfunction, and pathological neovascularization	EGFP-labeled endothelial cells and developing vascular network	Investigate the anti-angiogenic activity of 240 natural products	Chen et al. (2018)
AB strain + cnnm2 and nt5c2 knockout	cnnm2 and nt5c2	Blood Pressure	Altered blood flow parameters	To identify the most likely causal genes associated with BP	Chen et al. (2018)
dyrk1aa col4a1 rock2a/b loss of function	yrk1aa col4a1 rock2a/b	Cranial hemorrhage	Spontaneous cranial hemorrhage	Role of dyrk1aa, col4a2, and rock2a/b in vascular stability	Burgess and Burton (2023)
Redhead (rhdmi149)	p21-activated kinase 2a (pak2a)	Angiogenesis, regulation of cytoskeletal structure, endothelial cell migration, and contractility	Severe hemorrhage	Role of Pak2a	Buchner et al. (2007)
rasa1a and ephb4a mutants	rasa1a; ephb4a	Vein of Galen aneurysmal malformation (VGAM)	Failure of flow-dependent fusion of precursor vessels in the dorsal longitudinal vein, reproducing the genetic and structural features of VGAM.	Investigate flow-dependent endothelial mechanotransduction in VGAM pathogenesis and test pharmacological reversal via MAPK/PI3K modulation	Rodriguez-Pastrana et al. (2026)

Some transgenic backbones (e.g., fli1:EGFP, kdrl:GFP) also appear in Table 2 as general-purpose vascular imaging reporters; here they are shown only where paired with a specific disease-causing mutation or gene knockdown, consistent with this table’s focus on disease modeling rather than general vessel imaging.

### 7.1 Zebrafish models of genetic disorders

The zebrafish genome shares extensive homology with humans, including approximately 70% of protein-coding genes and 84% of disease-associated genes, highlighting the species’ utility as a model for studying human genetic disorders (Howe et al., 2013; Crouzier et al., 2021). To accomplish this, an increasing range of forward and reverse genetic techniques is available to generate new zebrafish models and identify novel genes regulating vascular development and pathology (Gore et al., 2012).

Among vascular regulators, the endothelial-secreted protein EGFL7 plays a critical role in angiogenesis and vasculogenesis. It promotes endothelial cell migration, proliferation, sprouting, and lumen formation, all of which are essential for proper vascular patterning and stability (Nikolic et al., 2010). In zebrafish, *egfl7* knockdown leads to defective endothelial cell migration and impaired vascular tube formation, resulting in abnormalities in vessel patterning and lumen development (De Mazière et al., 2008). EGFL7 also interacts with the microRNA miR-126, which is critical for vascular integrity, although their roles in vascular biology are partially distinct. These findings establish EGFL7’s indispensability in maintaining vascular architecture (Nichol and Stuhlmann, 2012).

Vascular anomalies are abnormal formations of blood vessels and can be categorized as either vascular malformations or hemangiomas (Holm et al., 2024). They could arise from aberrant proliferation of vascular endothelial cells or from structural defects in the vascular endothelium (Gokani et al., 2021). Zebrafish models that mimic human vascular malformations include arteriovenous malformations (AVMs), such as hereditary hemorrhagic telangiectasia (HHT), an autosomal-dominant disorder caused by mutations in the Transforming Growth factor-β (TGF-β) signaling pathway (Roman et al., 2002). The *violet beauregarde* (*vbg*) mutant carries a lesion in the *alk1* gene, resulting in severe edema and defective blood circulation, providing an effective model for type 2 HHT research (Roman et al., 2002; Driever et al., 1996). Mutations in the ras GTPase-activating protein Rasa1 cause AVMs in humans. Interestingly, *rasa1* mutants in zebrafish develop cavernous AVMs affecting the DA and caudal venous plexus, accompanied by altered blood flow dynamics and impaired *klf2a* expression, a flow-responsive transcription factor (Greysson-Wong et al., 2023). More recently, Martin-Valiente et al. developed complementary *rasa1a* and *ephb4a* zebrafish models to investigate vein of Galen aneurysmal malformation (VGAM), the most prevalent neurovascular malformation in neonates (Martin-Valiente et al., 2025). This work linked VGAM development to a failure of flow-dependent fusion of precursor vessels, showing that RASA1 deficiency destabilizes the endothelial response to blood flow and impairs flow-mediated MAPK and PI3K signaling. Pharmacological modulation of these pathways reversed the vascular phenotype in existing malformations, highlighting the zebrafish’s utility for both mechanistic dissection and therapeutic screening in AVM-related disease.

Another congenital vascular disorder modeled using zebrafish is Klippel–Trenaunay syndrome (KTS), characterized by capillary and venous malformations without any arterial defects, resulting in tissue hypertrophy (Wang, 2005). The syndrome is caused by upregulation and overexpression of AGGF1 (angiogenic factor with G-patch and FHA domain 1) due to a chromosomal translocation in the promoter region of the gene (Tian et al., 2004). AGGF1 protein is released outside of endothelial cells during angiogenesis and is required for endothelial tube formation. During zebrafish embryonic development, this gene is ubiquitously expressed, and overexpression results in increased angiogenesis, increased vein diameter, and abnormal PCV development. Contrarily, the knockdown of *aggf1* expression was shown to result in defective vasculogenesis, angiogenesis, and loss of venous identity (Chen et al., 2013).

Zebrafish have also been used to study cerebral cavernous malformations (CCMs), which are abnormally formed blood vessels composed of dilated, thin-walled vessels that are highly predisposed to hemorrhage (Chan et al., 2010). The disease usually involves the central nervous system (CNS) vasculature, but can also involve the systemic vasculature (Riant et al., 2010). Three conserved genes associated with disease onset have been identified in zebrafish: *krit1*, *ccm2*, and *pdcd10* (Chan et al., 2010). The *Santa* mutant strain was described as the zebrafish orthologue of the KRIT1 gene, and the *Valentine* mutant strain as the zebrafish orthologue of the *CCM2* gene (Chan et al., 2010). Another mutant strain, the *heart of glass*, was found to harbor a lesion in the receptor *heg1*, revealing remarkably similar dilated cardiac phenotypes to those of the *santa* and *valentine* mutants (Kleaveland et al., 2009). The abnormal, dilated, thin-walled, and closed vessels observed in zebrafish were reminiscent of those in human vessels with CCM. These findings indicate that zebrafish may be the first non-human model organism to phenotypically link all three genes associated with CCM. This provides a powerful tool for elucidating the genetic interactions of the CCM proteins.

Zebrafish have also been used to model other vascular disorders, such as von Hippel–Lindau (VHL) disease. This rare condition is caused by a mutation in the VHL tumor suppressor gene, which predisposes individuals to vascular tumors in multiple organs (Wilkinson and van Eeden, 2014). In zebrafish, vhl mutants exhibited markedly increased angiogenesis in embryonic blood vessels, beginning at two dpf (Wilkinson and van Eeden, 2014). Zebrafish *vhl−/−* mutants exhibit characteristics similar to VHL-associated vascular neoplasms observed in mammals (va et al., 2010). This indicates that *vhl* mutants could serve as a unique *in vivo* model for studying and understanding the underlying mechanisms associated with the disease.

### 7.2 Zebrafish models of endothelial dysfunction and flow-induced alterations

Shear stress is the force per unit area produced when a tangential force acts on a solid surface (Yuan and Reis, 2019). Flowing blood exerts wall shear stress (WSS) on the endothelial cells lining the vessel walls. Different blood flow profiles occur across the cardiovascular tree, and WSS varies across vascular segments. WSS can be categorized into different forms, such as pulsatile shear stress, mean shear stress, oscillatory shear stress, shear stress gradient, and laminar shear stress (Urschel et al., 2021; Papaioannou and Stefanadis, 2005). The force per unit area imposed by blood flow on the endothelium lining of blood vessels is referred to as WSS (Papaioannou and Stefanadis, 2005). On the other hand, pulsatile shear stress represents the variable component of shear stress caused by the pulsatile character of blood flow during the cardiac cycle (Takahashi et al., 2019). The mean shear stress refers to the time-averaged shear stress during the cardiac cycle (Liu et al., 1999). Oscillatory shear stress arises when the direction of shear stress alternates throughout the cardiac cycle (Storch et al., 2018) usually occurring at vessel branching and curvatures. Laminar shear stress forms in areas of smooth, undisturbed blood flow, which is often associated with straight blood artery segments and small capillaries (Pan, 2009; Dhawan et al., 2010). Changes in shear stress along the length of a blood artery are referred to as shear stress gradient (Ju et al., 2018).

There are several shear stress-sensing mechanisms in blood vessels. One important organelle is the cilium, a microtubule-based cellular protrusion observed on numerous cell types, including blood vessel endothelial cells (Gupta et al., 2022). Cilia play a crucial role in cellular signaling and mechano-transduction, serving as indicators of vascular development and integrity (Kallakuri et al., 2015). Moreover, they help detect and respond to mechanical stresses, such as fluid shear stress arising from blood flow, thereby regulating vascular homeostasis and architecture in Zebrafish (Yin et al., 2021). Several studies have also shown that endothelial cilia contribute to blood flow sensing, a process crucial for controlling vascular function. Importantly, endothelial cilia also modulate signaling molecules that promote vascular wellbeing and healing. For instance, TGF-β signaling is improved by primary cilia, which are important for vasculature repair after injury (Eisa-Beygi et al., 2022). Additionally, in our previous work, we showed that cilia-linked markers have been used to identify vascular injury in Zebrafish, indicating their increasing utility in the diagnosis and prognosis of vascular-related diseases (Gupta et al., 2022). Dysfunction of primary cilia has been implicated in various vascular diseases. One such condition is atherosclerosis, in which malfunctioning cilia increase susceptibility to disease mechanisms and cause greater vascular damage (Wang et al., 2021).

The zebrafish can also serve as a model for studying lipid metabolism and atherosclerosis disorders. Disturbed hemodynamics (i.e., oscillatory WSS and low WSS) is directly related to the development of atherosclerosis and is among the most important biomechanical factors (Zhou et al., 2023). An atherosclerotic plaque typically begins at low WSS sites, and its development alters the local WSS topography. Low WSS promotes atherosclerosis, whereas high WSS avoids it. On the other hand, high WSS is associated with the emergence of vulnerable plaque phenotypes as plaques advance (Zhou et al., 2023). Different forms of shear stress may trigger focal alterations in plaque composition and spatial variations in plaque rupture, atherosclerosis development, and susceptibility to thrombus formation. Zebrafish embryos can also be used to model several processes involved in atherosclerosis pathogenesis, including apoptosis, impaired vascular repair, and regeneration. Studies have shown that feeding zebrafish a high-cholesterol diet leads to significant hypercholesterolemia and lipoprotein oxidation, making zebrafish a potentially attractive model for studying developmental mechanisms of atherosclerosis. This chronic inflammatory disorder develops in arteries exposed to disturbed blood flow patterns and low wall shear stress. This mechanical environment activates endothelial cells, leading to inflammation, apoptosis, and the development of atherosclerotic lesions (Serbanovic-Canic et al., 2017). Lee et al. reported that the magnitude of wall shear stress in zebrafish vasculature is comparable to that in humans (Lee et al., 2015), An association between low oscillating shear stress and early atherosclerosis was also reported in a hypercholesterolemic zebrafish model (Lee et al., 2015).

The metabolism of lipids and lipoproteins is remarkably similar between humans and zebrafish since the latter expresses all major lipid transporters, nuclear receptors, apolipoproteins, and enzymes that are involved in the process of lipid metabolism (Fang et al., 2014). Unlike mice, zebrafish were reported to express the ester transfer protein gene (*cept*). This gene is homologous to the human gene encoding a protein that transfers cholesteryl esters from antiatherogenic high-density lipoproteins (HDLs) to proatherogenic lipoproteins (VLDLs, IDLs, and LDLs). Emerging evidence suggests that zebrafish rapidly develop hypercholesterolemia due to retaining a *cetp* ortholog gene and their high sensitivity to the ingestion of excess cholesterol (Kim et al., 2011; Jin and Cho, 2011).

Recent work in the zebrafish model elucidated the link between lipid metabolism and angiogenesis regulation. Similar studies have also uncovered the role of *aibp, abca1, abcg1, mtp, apoB,* and *apoC2* in regulating angiogenesis in zebrafish. These findings pave the way for future studies in mammals that could enable new therapeutic approaches to modulate angiogenesis. Therefore, the establishment of zebrafish models for studying atherosclerosis is of great benefit for understanding the disease pathogenesis and for identifying potential therapeutic targets for preventing endothelial injury at atheroprone sites (Bowley et al., 2021).

To date, two genetic zebrafish models have been reported for studying hyperlipidemia: *ldlr* (low-density lipoprotein receptor) mutants and apolipoprotein C-II (apoc2), which were generated using CRISPR/Cas9-and TALEN-mediated genome editing, respectively (Liu et al., 2015; Liu et al., 2018). The *ldlr* knockdown mutants were generated for modeling hypercholesterolemia and vascular lipid accumulation, which are hallmarks of atherosclerosis and related vascular pathology (Liu et al., 2018). *Apoc2* zebrafish mutants displayed a phenotype like that of human patients with familial Apoc2 deficiency, in which mutants fed a normal diet developed hypertriglyceridemia. Injections of human APOC2 mimetic peptide or plasma from wild-type zebrafish were shown to rescue the hyperlipidemia phenotype in *apoc2* mutants (Liu et al., 2015). Of interest, one study showed that exposing adult zebrafish to a high-cholesterol diet can help mimic early atherogenesis and related *in vivo* vascular cell functions. These include vascular lipid accumulation, hypercholesterolemia, lipoprotein oxidation, and macrophage lipid uptake (Stoletov et al., 2009). Therefore, impaired lipid metabolism can be modeled in zebrafish using genetic and dietary approaches, making them important tools for screening and developing novel lipid-lowering compounds.

The transparency of zebrafish embryos at early stages provides a unique advantage for practical imaging of blood flow via conventional microscopy. This advantage enabled researchers to investigate the mechanobiological factors of blood flow in vascular development under normal and diseased conditions. These investigations revealed that several conserved pathways regulating cardiovascular development in zebrafish are also involved in cardiovascular disease, including BMP (bone morphogenetic proteins), TGFβ, Notch, and Wnt (Bowley et al., 2021). Zebrafish embryos and adults can be used as model organisms to investigate vascular responses to altered flow, including apoptosis. Also, they can be used to study the mechanisms of vasculature repair and regeneration, lipid metabolism and accumulation, and intracranial hemorrhage. Further, zebrafish embryos can be used to functionally screen shear stress-regulated genes and identify their roles in vascular mechano-responses (Souilhol et al., 2020). Recently, blood flow-induced Notch activation and endothelial migration improved vascular remodeling in embryonic zebrafish trunk (Weijts et al., 2018). Together, the differences in endothelial cells’ response to arterial and venous blood flow led to the formation of balanced vascular networks.

The zebrafish model was also used to study the effects of low oscillatory shear stress on vascular development. This includes pruning—endothelial cell polarization and migration toward the high-flow segment—of the brain vasculature (Chen et al., 2012) and sub-intestinal vein (SIV) (Kochhan et al., 2013). Also, the effect of high oscillatory shear stress on endothelial-to-mesenchymal transition in zebrafish aortic valves was studied (Heckel et al., 2015; Hove et al., 2003). Classic studies on zebrafish embryos investigated the effect of altered blood flow on the endothelial identity and the establishment of arterial-venous specification (Souilhol et al., 2020; Wang L. et al., 2011; Isogai et al., 2003). Reduced arterial blood flow velocity was shown to downregulate arterial markers and to lead to ectopic expression of venous markers. Other studies on zebrafish embryos lacking blood circulation showed that blood flow is not required for the gross anatomical patterning of the primary vasculature. However, it plays an important role in patterning the interconnections within the vascular network, and is therefore important for determining final arterial or venous identity in the functional network (Souilhol et al., 2020).

### 7.3 Zebrafish as a model for studying tumor angiogenesis

The discovery of anti-angiogenic and pro-angiogenic agents has a long history, particularly in the contexts of cancer treatment and regenerative medicine, driven by the goal of identifying and managing blood vessel development. Angiogenesis, the generation of new blood vessels from existing vasculature, is a critical process that supports tumor progression and facilitates tissue repair (Ferrara, 2011). Vascular Endothelial Growth Factor (VEGF) and some chalcones and derivatives have been identified as having pro-angiogenic activity (Chen et al., 2015). Briefly, in 1989, VEGF was first isolated and cloned by Napoleone Ferrara (Ferrara, 2011), which was the same factor identified by Harold Dvorak in an independent study (Senger et al., 1983). This discovery heralded a new understanding and regulation of pro-angiogenic factors and their role in endothelial cell proliferation (Mori et al., 2024) and permeability (Senger et al., 1986). In contrast, thalidomide, bevacizumab (Avastin) (Mukherji, 2010), kinase inhibitors (sunitinib and sorafenib) (Tamaskar et al., 2008), and natural products (curcumin and flavonoids) (Sagar et al., 2006) are characterized as anti-angiogenic. Anti-angiogenic agents aim to inhibit mechanisms that drive pathological neovascularization. For instance, thalidomide analogs inhibited endothelial cell growth and migration, regardless of their capacity to modulate the immune system (Dredge et al., 2002).

Zebrafish is a novel model organism in cancer research, particularly for investigating anti-angiogenic and pro-angiogenic agents. Zebrafish tumor models are generated using various approaches, including genetic manipulation, chemical carcinogenesis, and xenotransplantation, in which human tumor cells are implanted into zebrafish embryos or larvae. These models enable the study of tumor growth, angiogenesis, invasion, metastasis, and responses to anticancer therapies (Chen et al., 2021). The transparency of zebrafish embryos allows real-time visualization of tumor dynamics and angiogenesis, while their rapid reproduction and small size facilitate high-throughput screening of potential therapeutic agents (Tobia et al., 2011). This approach offers a cost-effective setup and the ability to study human tumor xenografts without immune rejection at early developmental stages (Chen et al., 2021). These include physiological differences from humans, particularly in temperature regulation and immune responses, and the lack of certain aspects of the mammalian tumor microenvironment, especially during early life stages (Chen et al., 2021). Despite these challenges, zebrafish provide a powerful tool for understanding cancer biology and testing potential therapeutics.

Recent studies have shown the potential utility of zebrafish in studying tumor angiogenesis, which is a key step in tumor growth and metastasis (Tobia et al., 2011). Different zebrafish models at various developmental stages have been established in cancer research, which has allowed for studying the mechanisms involved in angiogenesis and developing therapeutic regimens (Tobia et al., 2011; Tulotta et al., 2016). Both anti-angiogenesis and pro-angiogenesis compounds have been explored to understand their effects on tumor growth and vascular development.

Anti-angiogenesis therapies, such as those targeting the VEGF pathway, have been used in zebrafish to inhibit tumor vascularization and slow down tumor progression. These models are useful for screening compounds that affect angiogenesis, with the added benefit of real-time visualization of blood vessel formation in zebrafish embryos due to their transparency (Rust et al., 2019). However, as in mammalian models, anti-angiogenesis as a standalone approach in zebrafish has shown limited long-term effectiveness. Tumor adaptation mechanisms, such as activating alternative angiogenic pathways, can lead to resistance to VEGF inhibitors (Oguntade et al., 2021). As a result, combination therapies in zebrafish models, which incorporate anti-angiogenesis agents with other treatments like chemotherapy or targeted therapy, are becoming increasingly emphasized to improve therapeutic outcomes and overcome resistance (Oguntade et al., 2021).

The inhibition of vessel formation is considered an excellent target for cancer therapy (Cross et al., 2003; Serbedzija et al., 1999). Several studies have used the zebrafish model to screen molecules that affect blood vessel formation, including the angiogenesis inhibitors SU5416 and TNP470, both of which have also been tested in mammalian systems (Serbedzija et al., 1999). Also, the effects of dissolved low-molecular-weight compounds on angiogenesis were investigated. Generally, the effects of angiogenic compounds on the vasculature can be easily examined using staining and microscopic visualization. However, other recent assessment methods have also been developed. For instance, a novel zebrafish yolk membrane assay has recently been proposed as a tool for studying anti-angiogenic compounds by testing the effects of low- and high-molecular-weight inhibitors, including the TK-FGFR1 inhibitor (SU5402) and the FGF2 antagonist long-pentraxin-3 (PTX-3) (Nicoli et al., 2009). This novel assay differs from other angiogenesis assays in that it allows injection of the angiogenic stimulus in a well-defined, topically delivered manner to stimulate ectopic angiogenesis. This could enable screening various angiogenic compounds targeting a specific growth factor or receptor.

The zebrafish tumor angiogenesis model has some limitations. Organ and body size differ significantly from mammals, and zebrafish lack certain vascularized organs (e.g., lung and skin), which could impede antiangiogenic drug delivery and mode of action. As a result, the biological milieu and niche structure are different, which may interfere with drug transport, metabolism, and efficiency (Seth et al., 2013). Moreover, there are currently no adult immunopermissive zebrafish lines available, and options for orthotopic transplantation are limited. Most importantly, maintenance temperatures are different, as most mammalian tumors develop at 37 °C and zebrafish at 28 °C, making it challenging to study the process of xenograft tumor growth at the ideal temperature (Madar et al., 2013).

One important advantage of zebrafish is that it is an appropriate model for a high-throughput drug discovery platform (Mauro et al., 2019). For creating new anticancer treatments, targeting molecular pathways involved in tumor angiogenesis with specific medicines or inhibitors is critical (Maj et al., 2016). Anti-angiogenic therapies are medications that prevent tumors from developing their own blood vessels. With the approval of many anti-angiogenic medicines, most of which target VEGF signaling, the effectiveness of traditional chemotherapy has been boosted or even replaced, yielding superior patient outcomes (Maj et al., 2016). A study conducted by Chen et al. (2018) investigated the anti-angiogenic activity of 240 natural products using the zebrafish line Tg (*fli1a:* EGFP)y (Chen et al., 2018). The results indicated that mundoserone decreased ISV development in zebrafish embryos in a dose-dependent manner (Chen et al., 2018). This suggests that mundoserone may be an effective angiogenic inhibitor, acting via downregulating SLIT/ROBO1 and FGFR/PTPRB signaling and upregulating NOTCH1A signaling (Chen et al., 2018). Another study showed that tryptanthrin inhibits tumor growth in Tg (*fli1a:* EGFP)y zebrafish in a concentration-dependent manner by decreasing Dll4 protein expression while increasing *desma* and *Tnnt2c* gene expression (Lin et al., 2023). Additionally, the zebrafish can be employed as a predictive model for translational anti-angiogenic drug discovery, resulting in safer and more efficacious anti-angiogenic drugs and pro-angiogenic drugs entering clinical trials.

### 7.4 Zebrafish as a model for studying hypertension

Zebrafish represents a promising model for studying hypertension. The adult zebrafish action potential is similar to that of humans. For instance, the upstroke of the zebrafish action potential is guided by Na + channels and L-type Ca2+ channels, which are critical during the plateau phase (Vishnolia et al., 2020). Although zebrafish lack functional, slowly activating K+ currents, cardiac T-type Ca2+ currents indicate that adult zebrafish electrophysiology may be equivalent to the human embryonic phenotype (Nemtsas et al., 2010). The zebrafish model is a basic vertebrate model that offers the potential to profile cardiovascular medicines before commencing mammalian toxicity studies. A recent study compared the translational power of zebrafish with that of rats, dogs, and humans, using three model compounds: propranolol, losartan, and captopril. These compounds modulate two key systems that regulate cardiovascular function—the beta-adrenergic and renin-angiotensin systems (Margiotta-Casaluci et al., 2019). The results demonstrated that zebrafish cardiovascular responses to the model drugs were highly similar (more than 80%) to those in humans, both in direction and effect size. Given these findings and the benefits highlighted above, zebrafish appears to be an appropriate model system for early-stage cardiovascular and/or blood-flow research. A study by Vishnolia et al. using zebrafish as an animal model identified the most likely causative gene(s) for hypertension at the 10q24.32 locus (Vishnolia et al., 2020). They reported increased arterial pulse and elevated linear velocity in zebrafish larvae with *cnnm2* and *nt5c2* knocked down, using gene-specific splicing-modification transcriptional morpholinos.

Angiotensin II (AngII), the Renin Angiotensin System (RAS) pathway’s major effector, governs hemodynamic pressure overload-mediated cardiovascular pathology in mammals. Studies showed that zebrafish, like mammals, have a functional RAS (Hoffmann et al., 2018; Joshi et al., 2021). Furthermore, it was shown that AngII infusion increases salt absorption in zebrafish larvae (Kumai et al., 2014). It was demonstrated that long-term exposure to waterborne AngII causes cardiac remodeling in adult zebrafish (Filice et al., 2021). In zebrafish, AngII stimulates fibrotic gene expression, collagen deposition, cardiomyocyte enlargement, and cardiac cell proliferation. Thus, zebrafish can be a useful model for studying AngII-RAS pathway-mediated pathology (Joshi et al., 2021).

### 7.5 Zebrafish as a model for studying cranial hemorrhage

Zebrafish have proven instrumental in modeling cranial hemorrhage due to their genetic similarities to humans and the transparent nature of their embryos, enabling real-time observation of hemorrhagic events. For example, studies using zebrafish with mutations in the *dyrk1aa* gene have demonstrated spontaneous cerebral hemorrhages, providing insight into the role of this gene in vascular stability (Burgess and Burton, 2023). Another study identified *col4a1* mutations leading to vessel rupture, closely mimicking cerebral small-vessel disease in humans. Also, disruption of the *rock2a/b* genes in zebrafish caused cranial hemorrhage and vascular malformations, highlighting the role of RhoA signaling in endothelial barrier function. The zebrafish *pak2a* mutant is a well-established model for studying cranial hemorrhage, as loss of *pak2a* disrupts endothelial stability and leads to reproducible vascular fragility in the developing cranial vessels (Buchner et al., 2007). These models also facilitate high-throughput screening for potential therapeutics, making zebrafish a robust system for studying the mechanisms and treatment of cranial hemorrhages (Burgess and Burton, 2023).

Therefore, zebrafish larvae provide a unique platform for drug screening, enabling researchers to identify neuroprotective chemicals immediately after cranial hemorrhage happens. Mammalian models of cranial hemorrhage have increased our understanding of disease pathways. However, their use as a pre-clinical trialing method for candidate drugs has so far been very limited in terms of clinical translation (Crilly et al., 2019). As a result, alternative pre-clinical methodologies for identifying and testing therapeutic molecules are necessary (Selim et al., 2018; Withers et al., 2020). Previously, zebrafish brain hemorrhage models have been used to identify chemicals that can reduce cerebral hemorrhage (Yang et al., 2017). These findings identify miconazole as an inhibitor of brain hemorrhages in zebrafish by decreasing matrix metalloproteinase 9 (MMP9)-dependent vessel rupture (Yang et al., 2017). This sheds light on the potential of zebrafish phenotype-based screens to discover new drug candidates and chemical probes for hemorrhagic stroke. To date, zebrafish appear more applicable than mammals for the drug development process in hemorrhagic stroke, since brain-specific bleeds occur spontaneously in larvae. Hematomas can also be easily detected using noninvasive microscopy, allowing reasonably quick screening (Crilly et al., 2022).

Beyond drug discovery, the transparent nature of the zebrafish also enables the deep mechanistic study of factors underpinning vessel integrity, such as primary cilia. For instance, in our previous work (Gupta et al., 2022). We used a larval model to demonstrate the critical role of primary cilia in cerebral endothelial cells as mechanosensors essential for endothelial barrier maintenance. We showed that increased shear stress due to increased heartbeat leads to physical loss of ciliary structure, resulting in compromised cerebrovascular integrity that manifests as haemorrhage in the brain vasculature. The role of cilia in preserving the integrity of cranial vessels was confirmed by genetic disruption of cilia, which again resulted in loss of vascular integrity and brain haemorrhage. More recent work in zebrafish has also shown that primary cilia in endothelial cells are located on the basolateral side (Prabhudesai et al., 2026). This new discovery suggests that endothelial basolateral cilia can interact with other brain cell types to promote vascular stability. In this recent publication, pak2a-PDGF-BB-heparan sulfate proteoglycan signaling was implicated in promoting recruitment of pericytes to facilitate vascular stability. These findings underscore the indispensable role of the primary cilium in preserving endothelial barrier function against hemodynamic stress, further validating the zebrafish as a platform for dissecting the fundamental causes of hemorrhagic disease.

## 8 Comparative evaluation of zebrafish and other animal models: strengths, limitations, and translational considerations

A variety of animal models have been utilized to investigate vascular development and vascular diseases, each offering distinct advantages and limitations (Table 4). Zebrafish have emerged as a powerful vertebrate model for studying vascular development and vascular-related diseases. Zebrafish have been widely used to investigate early-stage atherosclerosis, thrombosis, diabetic vasculopathy, myocardial injury, heart failure (HF), cardiomyopathies, arrhythmias, congenital heart disease (CHD), vascular malformations, endothelial dysfunction and flow-induced alterations, hypertension, cranial hemorrhage, and tumor angiogenesis (Weijts et al., 2018; Fang et al., 2011; Mauro et al., 2022; Hwang et al., 2023).

**TABLE 4:** Comparison of zebrafish and other animal models used in vascular development and vascular disease research.

Animal model	Primary vascular applications	Disease models	Human relevance	Genetic manipulation tools	Imaging capability	Throughput and cost	Major strengths	Limitations	References
Zebrafish (*Danio rerio*)	Vascular development, vascular remodeling, vascular regeneration, live imaging, vascular toxicology, and drug screening	Early-stage atherosclerosis, thrombosis, diabetic vasculopathy, myocardial injury, HF, cardiomyopathies, arrhythmias, CHD, vascular malformations, endothelial dysfunction and flow-induced alterations, hypertension, cranial hemorrhage, and tumor angiogenesis	Moderate, conserved vascular pathways, but with non-mammalian physiology	CRISPR/Cas9, Tol2 transgenesis, ENU mutagenesis, and Morpholinos	Real-time whole-organism imaging in transparent embryos and larvae	Very high throughput and low cost	Live vascular imaging, rapid development of transgenic lines, and high-throughput drug screening	Physiological and anatomical differences compared to mammals; developmental-stage constraints; limited models for studying advanced hemodynamics, advanced atherosclerosis, coronary artery disease, and vascular interventions; translational challenges; and validation needs in mammals	Weijts et al. (2018), Fang et al. (2011), Mauro et al. (2022), Hwang et al. (2023)
Medaka (*Oryzias latipes*)	Vascular development, vascular morphogenesis, comparative vascular genetics, vascular toxicology, and drug screening	Cardiomyopathies, arrhythmias, myocardial injury/fibrosis, CHD, vascular malformations, cardiotoxicity, and metabolic cardiovascular disorders	Moderate, conserved cardiovascular development and signaling pathways, but with limited mammalian disease relevance	CRISPR/Cas9, Tol2 transgenesis, ENU mutagenesis, and Morpholinos	Real-time whole-organism imaging in transparent embryos and see-through adult strains	High throughput and low cost	Live developmental imaging, rapid development, well-developed vitelline circulation, high-throughput screening, and comparative genetics studies	Fewer established vascular disease models, more limited genetic and imaging resources, slower embryonic development compared to zebrafish, absence of pulmonary circulation, reduced translational validation compared with zebrafish, and density-dependent phenotypes	Fujita et al. (2006), Ito et al. (2014), Kirchmaier et al. (2015), Higashikuse et al. (2019)
Chicken Embryo (*Gallus*)	Vascular development, angiogenesis, vascular patterning, and vascular toxicology	Developmental vascular defects, vascular malformations, tumor angiogenesis, and vascular toxicity	Moderate, highly conserved embryonic cardiovascular development and angiogenesis	CRISPR/Cas9, viral vectors, and electroporation	Real-time imaging through the CAM and embryo windowing techniques	High throughput and low cost	Accessible embryonic vasculature and angiogenesis assays	Limited genetic manipulation, short experimental window, and restricted disease modeling beyond embryonic development	Tavakkoli et al. (2020), Chamoto et al. (2026)
Mouse (*Mus musculus*)	Cardiovascular development, vascular remodeling, endothelial biology, vascular inflammation, and preclinical studies	Atherosclerosis, hypertension, thrombosis, stroke, aneurysm, diabetic vasculopathy, MI, heart failure, cardiomyopathies, and CHD.	High, conserved cardiovascular physiology, vascular signaling pathways, and disease mechanisms in humans	CRISPR/Cas9, knock-in, knockout, Cre-LoxP, and transgenesis	Intravital microscopy and advanced multimodal imaging, including ultrasound, MRI, CT, PET, and fluorescence imaging	Moderate throughput and moderate-to-high cost	Extensive genetic toolkit, well-characterized disease models, and high physiological relevance to human vascular disease	Higher cost and lower throughput, with some species-specific differences in disease progression	Shamsuzzaman et al. (2024), Wang et al. (2018), Cui et al. (2026)
Pig (*Sus scrofa*)	Vascular physiology, coronary vascular biology, vascular intervention, cardiovascular imaging, and translational research	Atherosclerosis, coronary artery disease, thrombosis, aneurysm, MI, HF, restenosis, and peripheral artery disease	Highly conserved cardiovascular anatomy and physiology closely resemble those of humans	CRISPR/Cas9, SCNT, and transgenesis	Advanced multimodal imaging, including ultrasound, MRI, CT, PET, OCT, IVUS, and angiography	Low throughput and very high cost	Human-like cardiovascular anatomy and physiology, clinical-grade imaging, and suitability for translational and device-testing studies	High maintenance costs, long generation times, limited genetic resources, and low experimental throughput	Lee et al. (2018), Brenner et al. (2021)
Non-human Primate	Vascular physiology, cardiovascular biology, disease modeling, therapeutic evaluation, and translational research	Atherosclerosis, hypertension, stroke, coronary artery disease, thrombosis, metabolic vascular disorders, and vascular aging	Very high, closest to human cardiovascular physiology	CRISPR/Cas9, viral vectors, and transgenesis	Advanced multimodal imaging, including ultrasound, MRI, CT, PET, and angiography	Very low throughput and extremely high cost	Closest cardiovascular physiology and disease manifestations to humans, supporting advanced translational research	Ethical constraints, extremely high costs, long generation times, and limited accessibility	Babici et al. (2020), Laila et al. (2022)

CHD, congenital heart disease; HF, heart failure; HCM, hypertrophic cardiomyopathy; MI, myocardial infarction; ENU, N-ethyl-N-nitrosourea; SCNT, somatic cell nuclear transfer; CAM, chorioallantoic membrane; OCT, optical coherence tomography; IVUS, intravascular ultrasound.

Compared with other non-mammalian models such as medaka and chicken embryos, zebrafish benefit from a larger repertoire of genetic manipulation tools, numerous transgenic reporter lines, extensive genomic resources, and a broader range of established vascular disease models (Hwang et al., 2023; Gierten et al., 2020; Tavakkoli et al., 2020). Their external fertilization, rapid embryonic development, optical transparency, and high fecundity enable real-time visualization of vascular morphogenesis and facilitate large-scale genetic and pharmacological screening approaches that are difficult to achieve in most vertebrate models. Compared with mammalian systems, zebrafish provide unparalleled opportunities for live imaging of vascular development at cellular resolution and offer substantially lower maintenance costs and higher experimental throughput, making them one of the most widely used animal models in vascular biology research (Mauro et al., 2022).

Despite these advantages, zebrafish possess several limitations that should be considered when interpreting experimental findings. Unlike mammals, zebrafish possess a simplified, two-chambered heart, lower systemic blood pressure, and single circulation, lacking a separate pulmonary circuit (Poss et al., 2002; Hu et al., 2001). These physiological and anatomical differences limit their utility in modelling complex human cardiovascular conditions such as myocardial infarction, atherosclerosis, and pulmonary hypertension (Asnani and Peterson, 2014) and make zebrafish less suitable for modelling disturbed hemodynamics, plaque rupture, and complex coronary artery disease compared with mammalian models. Genetic interpretation carries its own caveats: the teleost-specific whole-genome duplication can produce functional redundancy and genetic compensation that masks gene function, and discrepancies between morpholino-mediated knockdown studies and stable CRISPR-generated mutants—with some vascular phenotypes absent or substantially attenuated in mutant lines—have been attributed to off-target effects, genetic compensation, and differences in the extent and timing of gene suppression (Kok et al., 2015; Rossi et al., 2015; Van Gils and Vanakker, 2019; Rouf et al., 2023). Together, these anatomical and genetic caveats suggest that zebrafish findings do not always translate directly to mammalian systems, and key results should be validated using complementary genetic approaches and, where possible, in mammalian models.

While medaka share zebrafish’s optical transparency and amenability to genetic manipulation, they lag in translational resources. Medaka have been used to study cardiomyopathies, arrhythmias, myocardial injury and fibrosis, congenital heart disease, vascular malformations, cardiotoxicity, and metabolic cardiovascular disorders (Fujita et al., 2006; Ito et al., 2014; Kirchmaier et al., 2015; Higashikuse et al., 2019). However, they exhibit reduced regenerative capacity and currently possess fewer vascular-specific transgenic lines and disease models (Ito et al., 2014). This comparatively limited availability of genetic, imaging, and disease-modeling resources has led to zebrafish becoming the most widely used fish model for vascular biology research (Lin et al., 2016).

Moving beyond fish models, the chicken embryo offers a relatively inexpensive platform for investigating vascular development and has been widely used to study developmental vascular defects, vascular malformations, tumor angiogenesis, and vascular toxicity (Tavakkoli et al., 2020; Chamoto et al., 2026; Shekatkar et al., 2024). The accessibility of the embryo and the chorioallantoic membrane (CAM) assay make it particularly well-suited for studying angiogenesis and vascular responses to therapeutic interventions (Wan et al., 2024; Ribatti, 2016). Nevertheless, the model is constrained by limited genetic manipulation tools, a relatively short experimental window, and restricted applicability for modeling chronic vascular diseases beyond embryonic development (Kennedy et al., 2022).

Among mammalian systems, mice remain the most widely used model for vascular biology and disease research due to the availability of sophisticated genetic approaches, including conditional, inducible, and tissue-specific gene manipulation (Cleaver, 2021; Doetschman and Azhar, 2012). Mouse models have been instrumental in elucidating the molecular mechanisms underlying atherosclerosis, vascular inflammation, hypertension, thrombosis, coronary artery disease, and vascular aging (Xu, 2004; Shamsuzzaman et al., 2024; Wang et al., 2018; Cui et al., 2026; Lee et al., 2018; Brenner et al., 2021). Their greater physiological and anatomical similarity to humans enhances the translational relevance of experimental findings relative to zebrafish (Vedder et al., 2020). However, live imaging of vascular development is technically more challenging in mice, and large-scale genetic and drug screening studies are considerably more labour-intensive, time-consuming, and expensive than in zebrafish.

Larger mammalian models, including pigs and non-human primates, remain indispensable for investigating advanced cardiovascular pathologies, vascular interventions, device testing, and translational therapeutics, since their cardiovascular anatomy, vessel size, and hemodynamic parameters closely resemble those of humans (see Table 4) (Suzuki et al., 2011; van der Velden et al., 2022). However, these models carry substantial financial costs, longer generation times, lower experimental throughput, and increased ethical considerations, limiting their widespread use for mechanistic studies and high-throughput screening (Sridharan et al., 2023; Houthoofd et al., 2026).

There is, therefore, no single best model for studying vascular development and vascular-related disease. Zebrafish are strongest for conserved-pathway discovery, live imaging, and high-throughput screening. In contrast, pigs and non-human primates are necessary when the question depends on human-scale anatomy, device delivery, advanced pathology, or late preclinical safety and efficacy, with mice, medaka, and chicken embryos occupying complementary positions between these extremes. Zebrafish occupy a unique niche between simple developmental models and mammalian disease models: their physiological differences limit direct replication of human vascular pathology, but they remain an invaluable tool for uncovering fundamental mechanisms of vascular biology, identifying novel disease-associated pathways, and screening candidate therapeutics. Integration of zebrafish discoveries with validation in mammalian systems will continue to strengthen the translational impact of vascular research.

## 9 Conclusion

In conclusion, the zebrafish model is a tremendous asset for advancing our understanding of vascular development and disease. These features make it an ideal platform for real-time imaging and experimental manipulation of the vasculature. Zebrafish have provided important insights into the molecular mechanisms that orchestrate vascular development, providing a platform for unraveling the complexity of angiogenesis and vasculogenesis. Furthermore, their genetic adaptability facilitates the modeling of vascular disorders, allowing researchers to explore pathogenic mechanisms and screen prospective therapeutic approaches. The zebrafish continues to play an essential role in advancing vascular biology research as a flexible, cost-effective model organism, ultimately enabling the development of novel approaches to identify and treat vascular diseases in humans.
